# The Site-Specific Amino Acid Preferences of Homologous Proteins Depend on Sequence Divergence

**DOI:** 10.1093/gbe/evy261

**Published:** 2018-11-29

**Authors:** Evandro Ferrada

**Affiliations:** Center for Genomics and Bioinformatics, Faculty of Science, Universidad Mayor, Camino La Pirámide 5750, Huechuraba, 8580745, Santiago, Chile

**Keywords:** site-specific amino acid preference, thermodynamic stability, biophysical models of protein stability, amino acid substitution models, protein evolution

## Abstract

The propensity of protein sites to be occupied by any of the 20 amino acids is known as site-specific amino acid preferences (SSAP). Under the assumption that SSAP are conserved among homologs, they can be used to parameterize evolutionary models for the reconstruction of accurate phylogenetic trees. However, simulations and experimental studies have not been able to fully assess the relative conservation of SSAP as a function of sequence divergence between protein homologs. Here, we implement a computational procedure to predict the SSAP of proteins based on the effect of changes in thermodynamic stability upon mutation. An advantage of this computational approach is that it allows us to interrogate a large and unbiased sample of homologous proteins, over the entire spectrum of sequence divergence, and under selection for the same molecular trait. We show that computational predictions have reproducibilities that resemble those obtained in experimental replicates, and can largely recapitulate the SSAP observed in a large-scale mutagenesis experiment. Our results support recent experimental reports on the conservation of SSAP of related homologs, with a slowly increasing fraction of up to 15% of different sites at sequence distances lower than 40%. However, even under the sole contribution of thermodynamic stability, our conservative approach identifies up to 30% of significant different sites between divergent homologs. We show that this relation holds for homologs of diverse sizes and structural classes. Analyses of residue contact networks suggest that an important determinant of these differences is the increasing accumulation of structural deviations that results from sequence divergence.

## Introduction

A variety of biophysical and evolutionary forces affect the process of amino acid substitution in protein sequences. Among these forces are the maintenance of molecular structure and function (DePristo et al. 2005); thermodynamic stability (Tokuriki and Tawfik 2009); purifying selection against aggregation and misfolding (Drummond and Wilke 2008); protein–protein interactions (Levy et al. 2012); and protein expression (Rocha and Danchin 2004; Drummond et al. 2005). Over long-time scales, these forces manifest as biases in the amino acid composition of proteins sequences, or site-specific amino acid preferences (SSAP).

Evidence for the existence of SSAP comes from studies of multiple sequence alignments of protein homologs (Göbel et al. 1994; De Juan et al. 2013). Similar studies have revealed that the correlation of SSAP between amino acid positions contains information that is specific to distinct protein families and folds, can be used to reconstruct protein contacts (Morcos et al. 2011), are strongly associated to allosteric networks of residues responsible for function, and are often conserved over long evolutionary distances (Lockless and Ranganathan 1999; Shulman et al. 2004).

Accurate descriptions of the SSAP of a protein, or protein family, are essential for modeling molecular evolution. Indeed, models describing the *tempo* and *mode* of amino acid substitutions are the core machinery for the detection of divergent homologs and the construction of accurate phylogenetic trees (Yang 2014). The simplest of these models assumes that sites evolve independently of other sites, and that transition rates between different amino acids at a given site are proportional to the overall amino acid abundance in proteins (Dayhoff et al. 1978). Attempts to improve this model rely on the description of multiple parameters, often obtained from sequence data (Halpern et al. 1998), structural information (Koshi and Goldstein 1998), or more recently, from large-scale mutagenesis experiments (Bloom 2014). The improvement in phylogenetic fit obtained by these augmented models emphasizes the importance of incorporating site-specific information, as well as understanding the determinants of the amino acid preference of sites.

Even though it is clear that the SSAP of proteins vary across different structural folds, it is less clear whether the SSAP of two protein homologs is conserved, or whether it depends on sequence divergence. Answering this question has implications for the development of evolutionary models of protein evolution. One practical reason is that in the case of largely conserved SSAP, one would only need to estimate a single site-specific substitution model per fold (i.e., ≈2,000 models); in contrast, low conservation of SSAP would imply the derivation of models at much larger resolution (e.g., ≈15,000 protein families). A second important reason to understand the constancy of SSAP is to assess the degree to which the fixation of newly arising mutations in a population is influenced by genetic background, a phenomenon generally known as epistasis (Wolf et al. 2000). In particular, low/high degrees of intragenic epistasis are expected to translate into small/large differences between the SSAP of homologous proteins. Despite several theoretical and empirical studies, however, we currently know little about the strength and frequency of intragenic epistasis (Starr and Thornton 2016).

Pollock et al. drew interest to the problem of the constancy of SSAP by using extensive computer simulations of a model of the purple acid phosphatase protein (Pollock et al. 2012). Their results suggest that the SSAP of protein homologs is expected to change substantially as a function of mutations at other sites in the structure and that the surface accessibility of protein sites is an important determinant for the rate of change of SSAP. The authors validated their predictions by studying changes in thermodynamic stability of ferrodoxin. They showed that increasing divergent ferrodoxin homologs show consistent deviations in the reversibility of mutations (Pollock et al. 2012). Similarly, another study explored the effect of consecutive mutations under purifying selection for thermodynamic stability in a model of the lysine–arginine–ornithine binding periplasmic protein (Shah et al. 2015). The study showed that even at sequence distances of 30%, newly arising mutations can strongly depend on the fixation of previous mutations, or conversely, determine the fixation of future substitutions in a population. These initial observations have recently gained experimental support (Starr et al. 2018). 

Other researchers used mutagenesis experiments to explore differences in the SSAP of protein homologs. For instance, a large-scale study collected sequence data for a pair of closely related influenza nucleoprotein homologs and showed that sequence divergences of 6% translated into 3–15% changes in SSAP (Doud et al. 2015). Similarly, another mutagenesis study compared three TIM-barrel domain homologs and suggested that SSAP remained largely correlated at 30–40% of sequence divergence (Chan et al. 2017). Yet another study used a resurrected thioredoxin protein with 42% sequence divergence with respect to its extant (*Escherichiacoli*) homolog. The authors showed that exchanging the amino acid identity at 21 positions by the amino acids at the equivalent position in the thioredoxin homolog, led to strongly correlated changes in thermodynamic stability (Risso et al. 2015). These studies prompted authors to suggest that, in contrast to simulation results, the SSAP between homologs must be generally conserved at short, as well as long evolutionary distances (Ashenberg et al. 2013; Doud et al. 2015; Chan et al. 2017; Risso et al. 2015); however, see Pollock and Goldstein (2014). 

A limitation of previous studies, however, is that they only compared site-specific preferences in few, mostly related homologs (Doud et al. 2015; Chan et al. 2017). Similarly, due to the difficulty of some experimental assays, recent studies have only compared the SSAP at few equivalent positions, or relied on amino acid exchanges between homologs rather than evaluating the full distribution of SSAP per site (Ashenberg et al. 2013; Risso et al. 2015). Furthermore, and more importantly, results from these studies cannot always be directly contrasted, because the molecular trait under selection might have a differential impact on the SSAP. For instance, while some studies have focused on thermodynamic stability (Pollock et al. 2012; Ashenberg et al. 2013; Risso et al. 2015), other experiments were based on selection for a specific function (Doud et al. 2015; Chan et al. 2017).

Here, we seek to provide an alternative perspective on this problem by developing a computational procedure that allows us to estimate the SSAP of proteins based on changes in thermodynamic stability upon mutation. Despite its own limitations, computational predictions allow us to interrogate a large and unbiased sample of homologous structures, over the entire spectrum of sequence divergence, and under selection for the same molecular trait. Thus, our observations may help to clarify previous contrasting results between simulation and experiment, as well as provide clues about sequence and structure determinants responsible for differences in the SSAP of protein homologs. Our analyses show that computational predictions have reproducibilities similar to those observed in experimental measurements of replicate preference profiles; and can largely recapitulate the SSAP reported in a mutagenesis experiment. Analyses of a diverse sample of structure homologs reveal a monotonic increase in the difference of SSAP as a function of sequence divergence. Although our observations are conservative, they generally support conclusions from previous mutagenesis studies using closely related homologs, but also suggest that even under the sole contribution of thermodynamic stability, divergent homologs might have up to 30% of sites with significant differences. Analyses of residue contact networks suggest that the origin of these differences lies at the increasing accumulation of structural deviations that result from sequence divergence. Finally, we discuss the limitations and implications of our work.

## Materials and Methods 

### Prediction of Site-Specific Preference Profiles

**Fig. 1.**
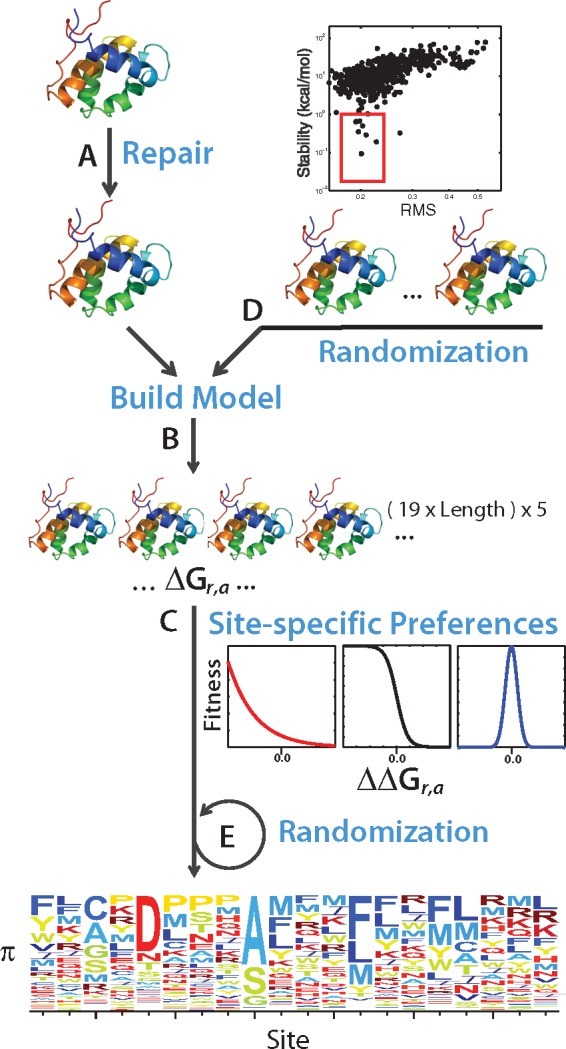
—Computational prediction of site-specific amino acid preferences. The procedure consists of three main steps. (*A*) We use three routines of the software FoldX to assess the quality of the input structure, reconstruct missing atoms and optimize thermodynamic stability. (*B*) The optimized structure is used as a template to build all (i.e., 19 times the protein length) possible single-mutant models. Each model is built five independent times. (*C*) Average changes in thermodynamic stability are used to calculate changes with respect to the stability of the input structure (ΔΔ*G_r,a_*); and then used to calculate SSAP using three models for the effect of thermodynamic stability on organismal fitness, according to equations (3) and (4). We simulate SSAP profiles by either (*D*) generating comparative models with random deviations in their atomic coordinates, and then repeating steps (*B*) and (*C*); or (*E*) by adding Gaussian noise to a multinomial distribution centered at the initial distribution of SSAP of each residue, and repeating the procedure for each residue in the SSAP profile (see Materials and Methods).

We predict changes in thermodynamic stability using the force field implemented in the software FoldX (Schymkowitz et al. 2005). The computational pipeline consists of three main steps (fig. 1). First, we use the FoldX routines *QualityAssessment* and *ReconstructSidechains* to identify erroneous side chains, and reconstruct residues with missing atoms. Reconstructed versions of the input structures are optimized using the routine *RepairPDB*, which carries out a local optimization by exploring sequential movements of residue side chains (fig. 1*A*). Second, we use the FoldX routine *BuildModel* to construct comparative models for all single possible mutations at every site of the protein. Error is estimated by modeling each mutant, five times. Third, we estimate the change in thermodynamic stability (ΔΔ*G*) caused by a mutation to residue *a*, at position *r*, with respect to the input structure (wt), as: $\Delta \Delta G=\Delta G_{r,a}-\;\Delta G_{r,wt}$. We use ΔΔ*G* values to derive the preference for amino acid at site *r*, according to three existing biophysical models. These models relate changes in thermodynamic stability to organismal fitness by estimating the effect of mutation on the protein's folding probability ($P_{f})$ (fig. 1*C*). The reason is that $P_{f}\;$ is inversely related to aggregation and toxicity, which reduce organismal fitness. Following several previous works (Pollock et al. 2012; Doud et al. 2015; Echave et al. 2015); we define the propensity of a site *r* to be occupied by the amino acid *a*, as:

(1) $$\pi_{r,a}=\frac{P_{f}(a)}{\sum_{j\in A}P_{f}(j)},$$

where A is the set of 20 amino acids. We use the 20-component vector $\vec{\pi_{r}}$, to represent amino acid preferences at site *r*; while the full SSAP profile, with 20 entries per site, is represented by the matrix π.

### Fitness Models Based on Protein Biophysics

The folding probability, $P_{f}$, in equation (1) can be calculated by using three types of biophysical models that summarize our current understanding of the relation between changes in thermodynamic stability (ΔΔ*G*) and folding (Echave and Wilke 2017; Bershtein et al. 2017). The so-called *threshold stability* model accounts for the existence of a threshold of minimal thermodynamic stability up to which a protein performs optimally. According to this model, mutations that increase stability have no effect on fitness, whereas mutations that reduce stability below the threshold have an unfavorable fitness effect, proportional to their decrease in stability. The threshold stability model can be obtained from Boltzmann statistics (Dill and Bromberg 2003); and has been derived in the context of protein fitness, independently, several times (Tokuriki and Tawfik 2009; Goldstein 2011; Wylie and Shakhnovich 2011). According to Boltzmann statistics, the folding probability of a protein can be calculated as:

(2) $$P_{f}=\frac{1}{1+e^{\Delta G/kT}}=\frac{e^{-\Delta G/kT}}{1+e^{-\Delta G/kT}}.$$

In our analyses, ΔG is the Gibbs free energy associated to the variant with amino acid *a* at site *r*, which can be expressed as: $\Delta G_{r,a}=\Delta \Delta G+\;\Delta G_{r,wt}$. Thus, substituting $\Delta G_{r,a}$, and approximating $\Delta G_{r,wt}\approx 0$, equation (2) becomes:

(3) $$P_{f}=\frac{e^{-\Delta \Delta G/kT}}{1+e^{-\Delta \Delta G/kT}}.$$

This model can be represented by a sigmoidal curve, such that mutations causing stabilizing changes (ΔΔG<0), are mostly neutral, whereas destabilizing mutations (ΔΔG>0) reduce fitness (fig. 1*C*). In our study, we used equation (3), and by exploring $\Delta G_{r,wt}$ as a function of error in structural data, we show that the approximation introduced in equation (2) does not affect our main conclusions (see Supplementary Material).

A second model of the effect of stability on folding is called *maximum stability* (Bloom et al. 2005; Echave et al. 2015; Echave and Wilke 2017). This model assumes that stabilizing mutations translate proportionally into a larger propensity to fold and can be generally expressed as:

(4) $$P_{f}={\alpha e}^{-\lambda {\left(\Delta \Delta G\right)}^{n}}.$$

For *n = *1 and λ=1/kT, we obtain the maximum stability model. The constant α vanishes in equation (1). Echave et al. (2015) studied the maximum stability model and used structural data to fit the parameter *λ*. They showed that under the normalization in equation (1), one can safely assume λ=1 (see eq. 13 and fig. 1 in Echave et al. 2015). For further details on the derivation of the maximum stability model, see Echave et al. (2015).

Using *n = *2 in equation (4), we obtain the *optimum stability* model (DePristo et al. 2005; Goldstein 2011; Shah et al. 2015; Echave and Wilke 2017). According to this model, $P_{f}$ is described as a Gaussian distribution, where fitness is optimal at the thermodynamic stability of the reference sequence, and both positive and negative deviations in stability, reduce $P_{f}$ (see Supplementary Material). Scripts to carry out the computational pipeline described above, using any of the three biophysical models, are provided in the Supplementary Material.

### Simulations of Replicate Profiles and Correlation between Profiles

In order to simulate replicates of a profile, with a given correlation with respect to the initial profile, we compared two alternative approaches. First, we used the routine *selection.randomize_xyz* of the software Modeller (Sali and Blundell 1993). We construct models of identical sequence with respect to the input structure, but introducing random deviations of ±1 to ±4 Å in their backbone atomic coordinates (fig. 1*D*). Structural deviations were measured as the structure root-mean squared deviation (sRMSD) over the *n* pairs of equivalent sites between the homologs, as:

(5) $$sRMSD=\;\sqrt{\frac{1}{n}\sum_{i=1}^{n}d_{i}}.$$

With *d_i_*, the Euclidean distance between equivalent sites. A pair of sites in two homologous structures is said to be equivalent if their $C_{\alpha }$ atoms are at distance of 3.5 Å or closer, after optimal superposition. In a second approach (fig. 1*E*), we simulated replicate profiles by generating a random sample of size *n* from a multinomial distribution ($\vec{m_{r}}$), centered at the amino acid preferences of site *r*: $\vec{\pi_{r}}$. In order to better control for the degree of error in $\vec{\pi_{r}}$, we introduce noise ($\xi_{r,a}$) distributed as a normal random variable centered at $m_{r,a}$: ${N[m}_{r,a},\sigma ].$ Then, the new SSAP for amino acid *a* at site *r*, is recomputed, as:

(6) $$\pi_{r,a}^{*}=\frac{m_{r,a}+\xi_{r,a}}{\sum_{j\in A}{[m}_{j,a}+\xi_{j,a}]}.$$

Replicate profiles with decreasing correlations with respect to the initial profile were obtained by setting *n *=* *100, and varying the parameter σ. Because both of these methods, the direct modeling of error in atomic coordinates and the introduction of noise, led to similar conclusions, we use the second approach, which is computationally more efficient, and should be less dependent on structural error across different input structures. The correlation between two profiles ($\pi^{a},\pi^{b})$, as well as the correlation between a profile and a replicate, was calculated using the Pearson correlation coefficient. In order to correct for multiple testing, we used the method of false discovery rate (FDR) (Benjamini and Hochberg 1995). MATLAB code to simulate profile replicates, determine correlations, and calculate FDR is provided as part of the Supplementary Material.

### Data Collection and Curation

We obtained GB1 protein sequencing data from Olson et al. (2014); and GB1’s SSAP profile using the program *dms_inferprefs.py* from the *dms_tools* software (Bloom 2015). In addition, we collected three additional structure data sets. In all cases we obtained data from SCOP (version 2.05, February 2015) (Murzin et al. 1995); filtered structures at the domain family level, larger than 50 amino acids, and with no DNA, RNA, or any other cocrystallized ligand in their original PDB entries. The first data set is composed of 175 homologous pairs of 100% sequence identity, solved by X-ray crystallography with resolutions ranging from 1.1 to 3.7 Å, and representative of the four main SCOP structural classes (supplementary table S1, Supplementary Material online). The second data set was obtained from the immunoglobulin binding family (SCOP family id: d.15.7.1) (supplementary table S2, Supplementary Material online). From this data set, consisting of 95 structure domains, we obtained a subset of 40 domains of high quality, solved by X-ray crystallography, with resolution ≤2.5 Å; or solved by nuclear magnetic resonance, and with initial thermodynamic stabilities lower than 0.5 kcal/mol. Finally, a third data set is composed of 124 pairs of representative homologs from the four main structural classes in SCOP, with lengths of 50–250 amino acids. This data set only included X-ray crystal structures, and selected alignments that span at least 95% of residues in each structure, and have sequence divergences of 0–100% (supplementary table S3, Supplementary Material online). As mentioned above, in all these data sets we used the software FoldX to assess structure quality, reconstruct incomplete side chains, and optimize thermodynamic stability using FoldX’s *repairPDB* routine. Lists with structure ids of each data set are provided as part of the Supplementary Material. 

### Structural Alignments and Structural Deviations

We collected 4,270 protein families from the 4 main structural classes of SCOP (version 2.05) (i.e., all-α, all-β, α + β, and α/β). We used the software TopMatch (Sippl and Wiederstein 2012), to perform pairwise structural alignments between pairs of protein domains that belonged to the same SCOP domain family, in an all-against-all manner. Because largely diverged proteins accumulate short insertions and deletions, we only studied alignments with sequence coverage larger than 95% of the structures under comparison. A pair of sites in two homologous structures is said to be equivalent if their $C_{\alpha }$ atoms are at distance of 3.5 Å or closer, after optimal superposition (Sippl and Wiederstein 2012). Structural deviation between pairs of structures was measured as the sRMSD over the equivalent $C_{\alpha }$-carbons (eq. 4). Alignments output by TopMatch maximize the number of equivalent sites between query and target structures. Because largely divergent homologs might be prone to misalignments, we identified alternative alignments with equally large coverage. All protein sequences, structures, SCOP ids, and structural alignments used in this work are provided as part of the Supplementary Material.

### Distance Metric and Exact Randomization Test

In order to identify sites with statistically significant amino acid preferences, we used the method described in Doud et al. (2015). The method is based on the Jensen–Shannon (JS) metric, a theoretic information measure for calculating the distance between discrete distributions (Lin 1991). The JS metric between the SSAP of two sites is calculated as the squared root of the JS divergence, and ranges between 0 and 1 for the minimum and maximum distance, respectively. Given two data sets, each consisting of a set of replicate preference profiles: [$\pi_{1}^{a},\;\pi_{2}^{a},\;\pi_{3}^{a},\;\ldots ,\;\pi_{n}^{a}]$ and [$\pi_{1}^{b},\;\pi_{2}^{b},\;\pi_{3}^{b},\;\ldots ,\;\pi_{m}^{b}]$; the method accounts for measurement error by comparing the RMSD of pairs of equivalent SSAP between profiles within and between data sets. To determine whether a site *r* has significantly different SSAP between two profiles ($\pi^{a}(r)$ and $\pi^{b}(r)$), the method first calculates the RMSD of the JS distance for all pairwise comparisons of SSAP at site *r*, in each separate data set (i.e., all pairwise comparisons of $\pi^{a}(r)$ over the *n* replicates of the $\pi^{a}$ data set; and all pairwise comparisons of $\pi^{b}(r)$ between the *m* replicates in the $\pi^{b}$ data set). This quantity is called RMSD_within_(*r*). Second, the method calculates the RMSD of the JS distance for all pairwise comparisons of SSAP at site *r*, for replicate profiles between the two data sets (i.e., *m*x*n* comparisons). This quantity is called RMSD_between_(*r*). Finally, the method calculates a normalized RMSD value for the site, or: RMSD_corrected_(*r*) =RMSD_*between*_(*r*) - RMSD_within_(*r*). A null distribution for RMSD_corrected_ for site *r*, can be obtained through an exact permutation test, by exchanging replicates of preference profiles between the $\pi^{a}$ and $\pi^{b}$ data sets, and recalculating RMSD_corrected_(*r*) (e.g., and detailed explanation, see Doud et al. 2015). MATLAB code that implements Doud et al.'s method, and the permutation test is provided in the Supplementary Material.

### Residue Contact Networks and Structural Analyses

To construct a residue contact network for the GB1 protein we estimated the fraction of times (*E*) two residues were observed in contact across all 1,064 single-mutant models. Two residues were defined in contact if at least one of their side chains atoms were at an Euclidean distance ≤3.5 Å; from each other (supplementary fig. S2, Supplementary Material online). Shortest path lengths were calculated using the Dijkstra algorithm, as implemented in MATLAB (MathWorks 2005). In addition, we constructed residue contact networks for 1mi0 and two of its Ig-binding homologs. In the case of this second network, we defined two residues in contact if any of their atoms were at a distance ≤3.5 Å; and distinguished among contacts per site (*r*), that were conserved ($C_{r}$, observed in both homologs); gained ($G_{r}$, only in the 1mi0's homolog); or lost ($L_{r}$, only in 1mi0 and not in the homolog). We calculated the fraction of rewired contacts ($f_{r}$) by distinguishing between the set of contacts at a given site *r*, in the first ($H_{r}^{a}$) and second ($H_{r}^{b}$) homolog, such as:

(7) $$f_{r}=1-J\left(H_{r}^{a},H_{r}^{b}\right)=\frac{G_{r}+L_{r}}{G_{r}+L_{r}+C_{r}},$$

where $J\left(H_{r}^{a},H_{r}^{b}\right)$ is the Jaccard index between the sets: $H_{r}^{a}$ and $H_{r}^{b}$. Amino acid volumes were obtained from the literature (Richards 1977; Wimley and White 1996); GB1 structure was illustrated using PyMol (DeLano 2002); surface accessibility was calculated using Naccess version 2.1.1 (Hubbard and Thornton 1993); and residue contact networks using the software Cytoscape (Shannon et al. 2003).

## Results

### Computational Prediction of SSAP

We start by implementing a computational procedure to estimate the SSAP of a protein. The procedure only uses structural information and is based on the computational modeling of single mutations followed by predictions of changes in thermodynamic stability (ΔΔ*G*) (fig. 1). SSAP can be estimated by using models of protein biophysics that relate ΔΔ*G* values to cellular fitness. The central idea is that mutations causing changes in thermodynamic stability affect a protein’s propensity to fold ($P_{f}$), a property strongly associated with fitness, via pathways leading to aggregation and toxicity (Drummond and Wilke 2008).

Three main alternative biophysical models have been proposed to explain the effect of stability on protein folding (Bershtein et al. 2017; Echave and Wilke 2017). The maximum stability model simply assumes that (de)stabilizing mutations contribute (un)favorably to folding. This model emerged from observations of strong selection for thermodynamic stability, and can be mathematically expressed by assuming that changes in stability result on an exponential decay of the protein’s folding propensity (eq. 4; fig. 1*C*, red curve). In contrast, the threshold stability model accounts for the existence of a critical level of thermodynamic stability up to which a protein performs optimally. This model arose from experimental evidence showing that proteins are often marginally stable and therefore, under some circumstances, the relation between stability and folding might follow a sigmoidal function (eq. 3; fig. 1*C*, black curve). Finally, the optimum stability model, accounts for constraints on functional performance. The main assumption of this model is that protein function is optimized at a particular value of thermodynamic stability, such as both stabilizing and destabilizing mutations are unfavorable. The model can be mathematically expressed as a Gaussian function centered at the protein’s optimal stability (eq. 4; fig. 1*C*, blue curve). We use equation (1) to compute site-specific preferences according to these three models (see Materials and Methods). SSAP for an entire protein are summarized by a matrix π that we call preference profile (or SSAP profile).

### Reproducibility and Comparison of Computationally Predicted Amino Acid Preference Profiles

Predictions of SSAP profiles are subject to error. In the case of SSAP profiles obtained experimentally, error is estimated by measuring replicate profiles for the same protein, multiple times. Then, differences between the profiles of two proteins are quantified by taking into account variation in the respective replicates (Doud et al. 2015). The computational pipeline presented above allows us to predict a protein’s SSAP profile based on structural data. However, because protein structures are rarely resolved more than once, we cannot use our computational pipeline to directly calculate replicate profiles from empirical data. We solve this problem by devising a method to simulate replicate profiles with an arbitrary degree of correlation with respect to the initial profile.

**Fig. 2.**
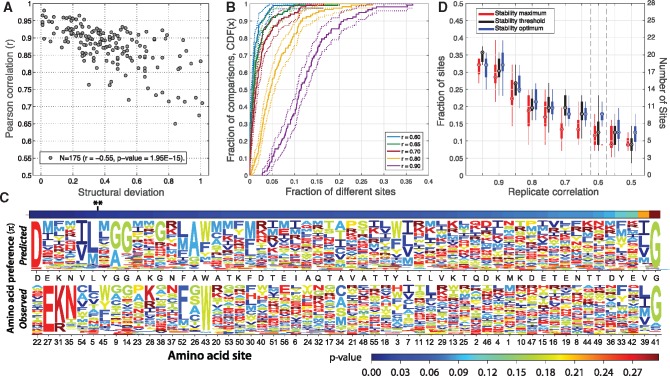
—The effect of structural deviations on the prediction of amino acid preferences and comparison of observed versus predicted preference profiles for the GB1 protein. (*A*) Association between structural deviations and the correlation coefficient for a set of 175 structure pairs with 100% sequence identity using the threshold stability model. Structural deviations were measured as the residual root-mean square error (sRMSD) between $C_{\alpha }$ carbons. Correlations between the predicted preference profiles, for each structure in the set of 175 pairs, were calculated according to the Pearson coefficient. (*B*) Cumulative distribution of the fraction of different sites per pairwise comparison versus the fraction of comparisons in the set of 175 structure pairs of identical sequences. Comparisons of SSAP profiles were carried out using the method of Doud et al. at a significance level of 5%. For each structure in the comparison, replicate profiles were simulated with Pearson correlation coefficients of 0.9 (purple), 0.8 (yellow), 0.7 (red), 0.65 (green), and 0.60 (blue). Data were calculated using the threshold stability model (cf. supplementary fig. S2, Supplementary Material online). (*C*) Top panel shows the GB1 observed profile, obtained from Olson et al. (2014). Bottom panel shows the predicted profile using GB1 crystal structure (PDB: 2gi9), and the pipeline described in figure 1. Profiles were calculated using the maximum stability model (cf. supplementary fig. S3, Supplementary Material online). Sites were sorted according to their *P*-value (**P-value <0.05). Sequence logos were constructed using the program *dms_plotlogo* (Bloom 2015). (*D*) Average fraction (and number) of sites with significantly different SSAP (*P*-value <0.05), between observed and predicted GB1's profiles, as a function of the average correlation coefficient among replicates. Predicted profiles were calculated using three alternative models for the effect of thermodynamic stability on fitness: Maximum (red); threshold (black); and optimum (blue) stability models (see Materials and Methods).

First, we reason that profiles predicted from structures of identical sequences are expected to have undetectable differences, or maximum similarity. We identified 175 pairs of structures with 100% sequence identity, and representative of the four main SCOP structural classes (supplementary table S1, Supplementary Material online). Second, for each of these structure pairs, we predicted preference profiles using our computational pipeline. Error in our predictions can arise from different sources such as variation in the quality of structural data, the intrinsic conformational flexibility of proteins, or from the limited accuracy of molecular force fields at predicting thermodynamic stability. In a pair of structures of identical sequences a combination of these factors should be reflected in the structural variation between pairs. Indeed, the set of structure pairs of identical sequences revealed a strong association between the correlation coefficient of their predicted SSAP profiles and structural variation, measured as the sRMSD (eq. 5) (Pearson’s *r* = ‒0.55, *P*-value = 1.95× 10^−15^) (fig. 2*A*).

Third, we devised a method to simulate replicate profiles with an arbitrary degree of correlation with respect to the initial profile. The method generates variation on an SSAP by introducing noise distributed as a normal random variable centered at the values of amino acid preferences of a site ($\pi_{r,a})$ (eq. 6). By repeating such procedure for each site of a profile, and for each of the 175 pairs of structures, we simulated replicates with correlations ranging from a Pearson’s *r* of 1.0–0.5. Finally, we used the method implemented in Doud et al. (2015) to identify the correlation between replicates so that the error rate for the detection of significant differences between SSAP in the 175 null pairs was approximately 5%. We found that, under the null hypothesis that homologs of identical sequence have identical SSAP profiles, replicate profiles with Pearson correlation coefficients of 0.60 led to an error rate of less than 5% in nearly 100% of comparisons (fig. 2*B*). Alternative biophysical models led to similar conclusions (supplementary fig. S2, Supplementary Material online).

We note that a Pearson correlation coefficient of 0.60 is a conservative definition. The reason is that structures of identical sequence do not necessarily lead to identical SSAP profiles (i.e., our null hypothesis is not always true) (fig. 2*A*). In particular, our control set of proteins (*N* = 175 pairs) includes structures of diverse folds and sizes, and with resolutions ranging from 0.8 to over 3.0 Å (supplementary table S1, Supplementary Material online). However, an overall Pearson correlation coefficient of 0.60 is not very different from replicate profiles obtained in large-scale mutagenesis studies, which show average correlations of 0.67 [e.g., 0.78 and 0.83 in human influenza nucleoprotein (Doud et al. 2015); 0.55–0.62 in human influenza hemaglutinin (Thyagarajan and Bloom 2014); 0.66 in Tn5 transposon (Melnikov et al. 2014)]. Indeed, 80% of the protein pairs in this control set shows at most 2% of sites misclassified as different (fig. 2*B*); and smaller correlation coefficients lead to similar differences (see below). Thus, in order to evaluate the similarity between SSAP profiles, we proceed as follows: For each profile under comparison we simulate pairs of replicates with an average Pearson’s correlation coefficient of 0.60, then we use the Doud et al. method to identify significant differences between SSAP profiles at a significant level of 5%.

### Computational Predictions Recapitulate the SSAP Observed in a Large-Scale Mutagenesis Study

We first tested the performance of the computational pipeline introduced above by focusing on a single protein structure, the domain B1 of the immunoglobulin-binding protein G (GB1) (Sauer-Eriksson et al. 1995). GB1 is 56 amino acid long with an α +β fold, for which large-scale mutagenesis (Olson et al. 2014), as well as extensive structure, kinetic and thermodynamic data are available (Sauer-Eriksson et al. 1995; Malakauskas and Mayo 1998; McCallister et al. 2000; Wunderlich et al. 2007).

We obtained mutagenesis data for all single mutations of GB1 from Olson et al. (2014), and used it to derive an SSAP profile. We refer to this profile obtained experimentally, as observed profile (fig. 2*C*, upper panel). In addition, we identified a crystal structure with 100% sequence identity with respect to the protein used in Olson et al. (2014) (PDB: 2gi9); and derived an SSAP profile through the computational procedure described above. We refer to the SSAP profile obtained computationally, as predicted profile (fig. 2*C*, lower panel). Finally, in order to compare observed versus predicted preference profiles, we simulated replicate profiles with an average Pearson’s correlation of 0.60, and identified sites with significant differences in amino acid preferences using the Doud et al. method (Doud et al. 2015).

The computational procedure implemented here identifies the most salient differences between observed versus predicted profiles (fig. 2*C*). A small fraction of sites shows a strong departure from their observed/predicted preferences. By definition, most of these sites are easily classified as having either very different (e.g., sites 22, 27, and 31); or very similar (e.g., 39, 41) SSAP. In addition, a large fraction of sites shows highly uniform amino acid preferences, and as in the case of large deviations in SSAP, their preferences are often predicted correctly. In contrast, sites with intermediate departures from uniformity are more difficult to predict. Most of these sites seem to have mild to strong biases toward a particular amino acid, often with physicochemical properties similar to the equivalent site in the observed profile (e.g., sites 5, 9, and 54) (fig. 2*C*).

As expected, the fraction of sites with significant differences in SSAP depends on the correlation coefficient of the simulated replicate profiles (fig. 2*D*). Replicate profiles with larger correlation coefficients (i.e., less measurement error), translate into a larger fraction of different sites. Replicates with an average Pearson’s correlation coefficient of 0.60 translate into six sites with statistically significant differences in SSAP (10%); and as suggested by our previous analysis, a smaller correlation coefficient lead to very similar differences (fig. 2*D*).

Interestingly, the use of alternative biophysical models for the effect of thermodynamic stability on fitness has little impact on the classification of sites with significantly different SSAP (fig. 2*D* and supplementary fig. S2, Supplementary Material online). The same six sites were identified as significantly different in all three models. The exponential model, however, seems to provide with a slightly better matching between predicted and observed profiles (fig. 2*C* and supplementary fig. S3, Supplementary Material online).

### Protein Sites Involved in GB1 Molecular Function Explain Unexpected Deviations in Amino Acid Preferences

Because the computational pipeline presented above is agnostic to the functional constraints experienced by GB1, and only can aspire to capture the contribution of thermodynamic stability in an isolated structure, we hypothesize that sites showing significant departures from the observed SSAP, are directly involved in the molecular function of GB1. We test this hypothesis using a crystal structure of GB1 bound to its natural ligand: The Fc domain of the immunoglobulin protein (PDB: 1fcc). This crystal structure provides direct information about the residues involved in GB1-binding function, as assayed in the mutagenesis experiment of Olson et al. (2014).

**Fig. 3.**
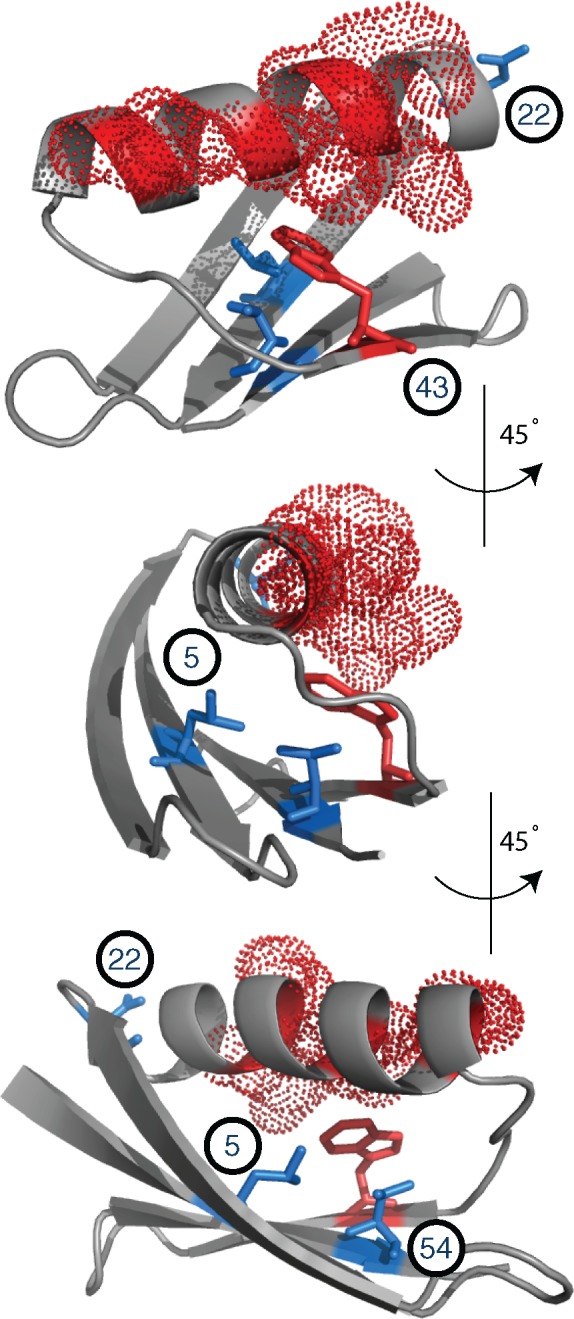
—Spatial distribution of sites with significant differences between observed versus predicted site-specific amino acid preferences of the GB1 protein. GB1 crystal structure (PDB: 2gi9) illustrating the spatial distribution of sites with significantly different SSAP detected through the comparison between observed and predicted preference profiles. Residues were colored according to *P*-values calculated according to the Doud et al. method. Residues with significant differences in SSAP, were classified as directly involved in GB1 binding interface (red): 27, 28, 31, 35 (dotted); 43 (sticks); and indirectly involved (blue): 5, 22, and 54 (sticks). Panels show the structure observed from three different angles of 45° of rotation around the vertical axis.

According to the crystal structure of GB1 in complex with the immunoglobulin subunit (Fc), the binding interface lies along the external face of GB1's alpha helix (fig. 3, residues in red); and encompasses five main residues that interact at distances closer than 3.0 Å with residues in the Fc chain. Notably, the binding interface includes sites 27, 31, and 35; which are among the sites most significantly deviated with respect to the observed GB1 preference profile (figs. 2*C* and 3). However, the other two residues, part of the binding interface at positions 28 and 43, are not among the sites with significant differences in SSAP detected through our procedure.

We collected additional evidence in support of the impact of GB1-binding function on sites with significantly deviated SSAP (see Supplementary Material). Firstly, previous studies suggest that site 22 is most likely involved in GB1's folding kinetics, which might explain why, in contrast to other sites showing significant deviations, preferences at position 22 deviate strongly in the predicted but not in the observed profile (fig. 2*C*). Secondly, we used all single-mutant structure models of GB1 to construct a residue contact network and demonstrated that sites with significant deviations in their amino acid preferences (i.e., 5, 54; or 5, 22, 54) are significantly closer than expected to residues involved directly in GB1-binding function when compared with any other sets of two or three randomly chosen residues in the structure (see Supplementary Material).

Overall, our results suggest that significant differences between predicted and observed SSAP profiles are strongly influenced by GB1's binding function, and therefore their deviation is unlikely to be explained by effects on thermodynamic stability alone. Overall, analyses of GB1's crystal structures reveal that out of the six sites (10%) with significant differences between the observed and predicted preference profiles, three sites (5%) are directly involved in the function under selection in the large-scale mutagenesis experiment; whereas the remaining three sites are significantly compromised by functional (and possibly kinetics) constraints. More importantly, our analyses suggest that thermodynamic stability can substantially contribute to the SSAP of proteins, and that the computational procedure implemented here can recapitulate such contribution.

### The SSAP of Homologous Proteins Depends on Sequence Divergence

In order to investigate differences in the SSAP of homologous proteins, we collected 95 homologous structures of the GB1 protein, which according to the SCOP classification, belong to the Ig-binding domain family (supplementary table S2, Supplementary Material online). GB1 homologs show an α +β fold, and despite of high-sequence divergence, they have a conserved function: The binding of the Fc immunoglobulin domain (Sauer-Eriksson et al. 1995). We aligned all possible pairs of structures in the GB1 family, selected 870 alignments that span on average 95% or more residues per structure, and have sequence divergences that range from 0 to over 90%. Finally, we applied our prediction pipeline to each structure; and used the Doud et al. method to estimate significant differences in the SSAP of pairs of profiles, as described above.

**Fig. 4.**
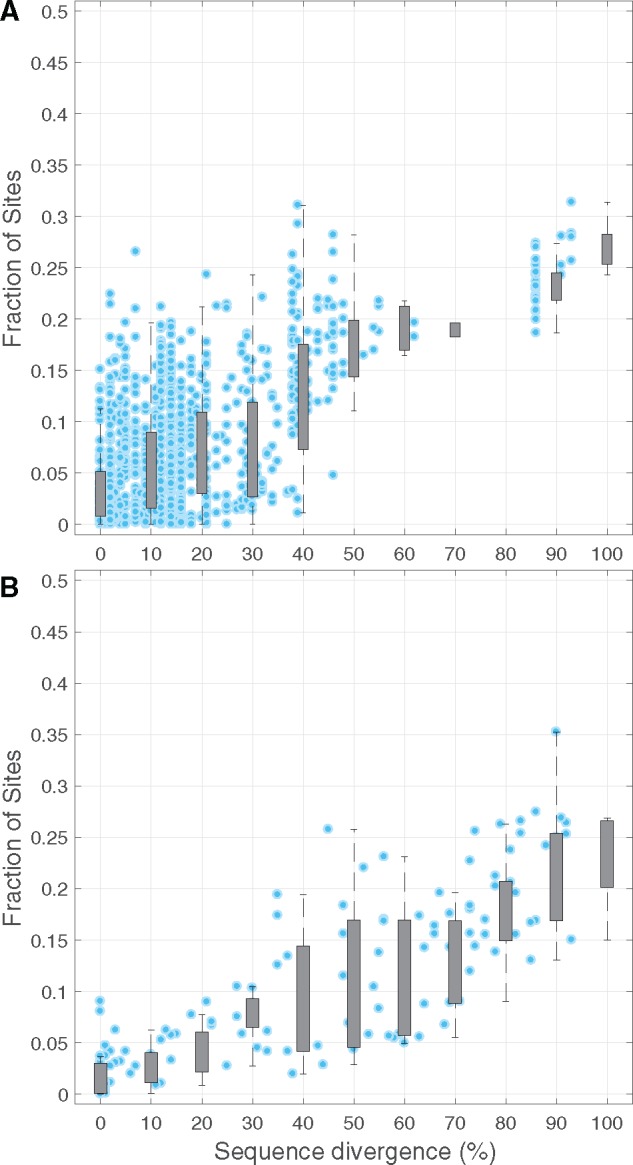
—The site-specific amino acid preferences of homologous proteins depend on sequence divergence. Fraction of sites with significant differences in SSAP between pairs of homologous structures, as a function of sequence divergence. (*A*) Comparisons of pairs of Ig-binding domain homologs. The Ig-binding domain family is composed of 95 structures. We compared 870 significant alignments that span on average 95% of residues in each structure and show sequence divergences that range from 0 to over 90%. (*B*) Comparisons of pairs of structurally diverse homologs. We compared 124 pairs of representative homologs of the 4 main structural classes in SCOP, spanning sequence divergences of 0–90%, and ranging from 50 to 250 residues in length (see Materials and Methods).

We first explored the average JS distance between profiles as a function of sequence divergence. In the case of Ig-binding domain homologs there is a monotonic increase of up to 5–10% (supplementary fig. S5*A*, Supplementary Material online). Increasing JS distances between profiles are accompanied by a substantial fraction of sites with statistically significant differences (fig. 4*A*). Consistent with findings reported by experimental studies, sequence divergences of up to 40% lead to changes of over 15% percent of sites (Doud et al. 2015; Chan et al. 2017). In addition, we also observe that increasing sequence divergences lead to larger fractions of sites with significant differences in SSAP. In the case of the Ig-binding domain family, these differences reach up to 25–30% of sites (fig. 4*A*).

In order to discard effects of structure quality on our results, we selected a subset of Ig-binding domain homologs with resolutions better than 2.5 Å, and conservative initial thermodynamic stabilities (see Materials and Methods). The effect of sequence divergence was also observed in this more conservative set of Ig-binding domains (supplementary fig. S6, Supplementary Material online). In addition, we tested alternative models for the effect of thermodynamic stability on fitness. In both, the optimum and threshold stability models we observed significant differences in SSAP reaching up to 30% of sites (supplementary fig. S7, Supplementary Material online).

Next, we wanted to explore whether the observed dependency of SSAP on sequence divergence is a peculiarity of the Ig-binding family, or whether it also holds for homologs of larger size and different folds. To answer this question, we identified 124 pairs of homologs across the four main structure classes of fold architectures compiled by SCOP (Murzin et al. 1995) (supplementary table S3, Supplementary Material online). For each of these pairs, we carried out structural alignments, predicted preference profiles, and identified significant differences using the method of Doud et al. as described above. As in the case of the Ig-binding family, changes in thermodynamic stability predict a significant monotonic increase in the fraction of sites with significant differences as a function of sequence divergence (fig. 4*B*). As observed earlier, alternative models for the effect of thermodynamic stability on fitness lead to similar results (supplementary fig. S7, Supplementary Material online). Our analyses revealed that the relation between sequence divergence and SSAP also holds for protein homologs of varying sizes, and diverse structural folds.

### Structural Deviations and the Amino Acid Preferences of Protein Homologs

**Fig. 5.**
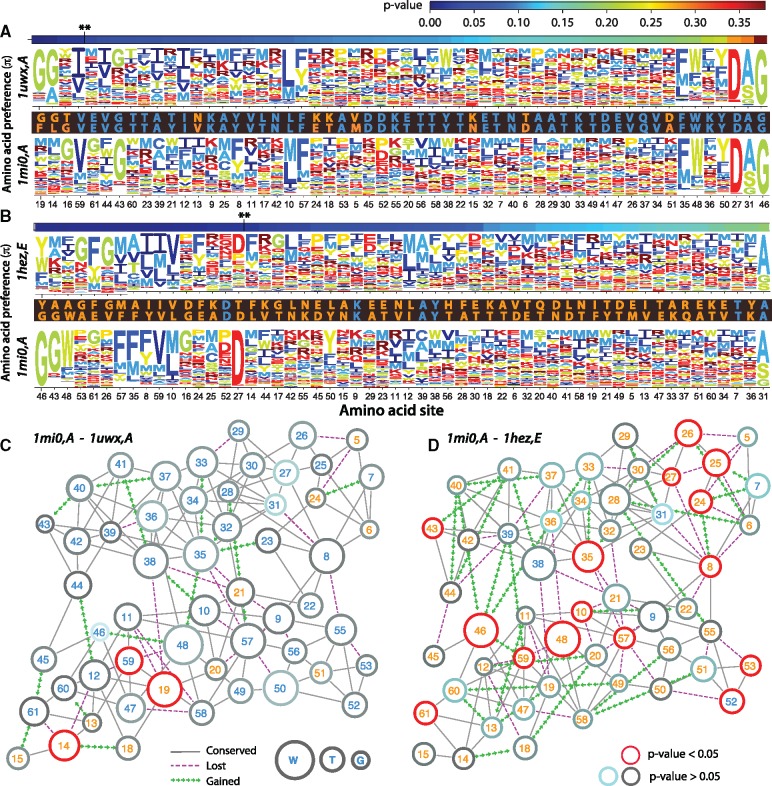
—Sequence divergence and the site-specific amino acid preference of pairs of immunoglobulin-binding protein homologs. We compared two pairs of Ig-binding protein homologs of known structure. The first pair (SCOP ids: 1mi0, A and 1uwx, A) has 23% sequence divergence (*A*, *C*). The second pair (SCOP ids: 1mi0, A and 1hez, E) has 89% sequence divergence (*B*, *D*). (*A*, *B*) Preference profiles were predicted according to the pipeline described in figure 1. Sites were sorted according to their *P*-values. Sequence alignments show substituted (orange) and conserved (blue) sites. Sequence logos were constructed using the program *dms_plotlogo* (Bloom 2015). (*C*, *D*) Residue networks were constructed by connecting any two at ≤3.5 Å, including side chain atoms. In the case of conserved residues, node size is proportional to the volume of amino acid side chains. If the residue was substituted, node size is proportional to the absolute difference in volume between the residues. Red circled nodes represent significant differences in SSAP; dark gray to light blue, decreasing nonsignificant differences. Node labels were colored according to their conservation between substituted (orange) and conserved (blue). Edges were classified as conserved (solid gray, observed in both homologs), gained (dashed green, only in the 1mi0's homolog), and lost (dashed purple, only in 1mi0 and not in the homolog).

Our analyses show that despite large structural diversity between pairs of homologs, there is a consistent degree of dissimilarity in the SSAP as a function of sequence divergence. Here, in order to gain insights on the structural determinants of these differences, we study a single member of the Ig-binding family (PDB: 1mi0), and two of its homologs at short (PBD: 1uwx), and long (PDB: 1hez) sequence distances. The homologous pair 1mi0/1uwx has a sequence divergence of 23%, with only three sites (5%) having significant differences in SSAP. In contrast, the homologous pair 1mi0/1heze has a sequence divergence of 89%, with 17 (29%) significantly different sites (fig. 5*A* and *B*). To gain insights on the molecular determinants of these differences, we constructed residue contact networks. We identified differences between equivalent sites by distinguishing between gained, lost and conserved contacts (edge coloring); substituted and conserved sites (node label colors); and in the case of sites with amino acids substitutions, we calculated absolute changes in volume (node size) (fig. 5*C* and *D*).

A first striking effect of sequence divergence is a substantial reorganization of the contacts between residues. The fraction of rewired contacts ($f_{r}$) at site *r* can be calculated as the ratio between gain and lost contacts, with respect to the total contacts (eq. 6; see Materials and Methods). Indeed, 25% of the contacts were either lost or gained in the pair of close homologs (16 gained, 23 lost, 118 conserved); whereas $f_{r}$ increased to 39% in the more divergent pair (31 gained, 36 lost, 105 conserved). Notably, most rewired regions of the networks seem strongly associated to sites with significant changes in SSAP. For instance, sites 24–27, as well as 46–59–48 in the 1mi0–1heze pair (fig. 5*D*). In addition, as suggested by the peripheral distribution of sites with nonsignificant differences in SSAP (e.g., sites 40, 41, 37, 33, 29 in fig. 5*B* and *D*); sites with on average larger surface accessibilities probably have more uniform SSAP. To test this hypothesis, we compared the entropy of the distribution of amino acid preference per site of buried (surface accessibility <25%) and exposed residues. We found that exposed sites have significantly more uniform SSAP (supplementary fig. S8, Supplementary Material online).

A third observation relates to the combined effect of contact density and the fraction of amino acid substitutions in the neighborhood of a site. The residue networks suggest that most significant differences are associated with absolute changes in the volume of substituted amino acids (e.g., sites 14, 19 in fig. 5*C*; sites 35, 46, 48 in fig. 5*D*); and/or the number of amino acid changes in neighboring sites (e.g., 14 in fig. 5*C*; site 57 in fig. 5*D*). A combination of these two effects can be seen at site 8, which in the closest pair of homologs (fig. 5*A* and *C*), is a conserved tyrosine, has a predominant preference for aromatic residues, no significant differences in SSAP, and interacts with other 9 (mostly conserved) sites, 2 of which were lost in the 1uwx homolog. In contrast, site 8 in the more divergent pair of homologs (fig. 5*B* and *D*), has been substituted for an isoleucine residue; four of its nine original contacts were lost, one gained, and consequently the site has significantly shifted its preferences.

In order to generalize our observations and quantify the impact of structural rearrangements on a site's amino acid preferences, we calculated the fraction of rewired contacts ($f_{r}$) between pairs of equivalent sites at position *r*. Our analyses included 14,460 comparisons of SSAP, collected from 124 homologous pairs, distributed across the four main structural classes in SCOP (supplementary table S3, Supplementary Material online). As expected, we find that pairs of sites with significant differences in SSAP reshape on average 40% of their contact shell, which is two times the expected fraction of changes at sites with nonsignificant differences in SSAP (Wilcoxon’s Rank Sum test, *P*-value = 1.7× 10^−92^).

**Fig. 6.**
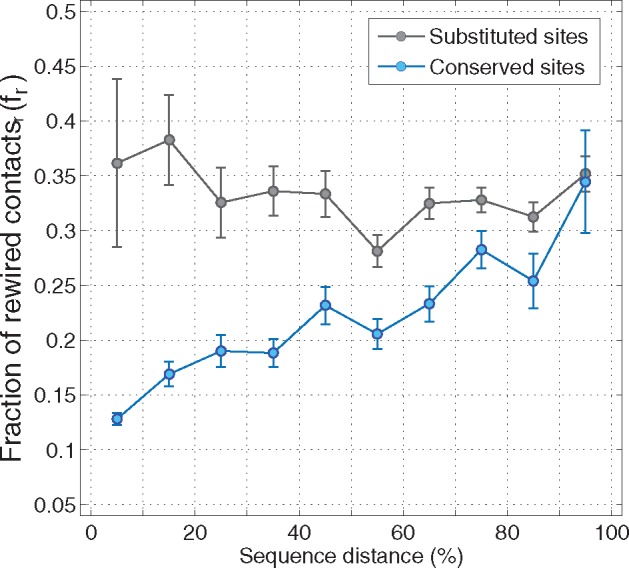
—The fraction of rewired contacts at conserved sites depends on the sequence distance between homologous proteins. Fraction of rewired contacts at equivalent sites ($f_{r}$) between homologous proteins at increasing sequence distances. All pairs of equivalent sites (14,460) were divided between substituted (gray; *N* = 6,134) and conserved (blue; *N *=* *8,326), and classified according to the sequence distance between the corresponding pair of homologs. For each site, in each sequence distance class, we calculate the average $f_{r}$; and estimate 95% confidence intervals using the bootstrap (*n *=* *10,000).

Most changes in the number of contacts at a particular site must be due to both, the amino acid substitution at the site, as well as the substitutions at other positions in the structure. In order to tease these factors apart, we distinguished between sites that have been substituted and sites with conserved amino acids, and calculated $f_{r}$ as a function of sequence divergence. Our results reveal that, regardless of the sequence divergence between homologs, substituted sites experience relatively constant $f_{r}$ values, with an average of 30–40% of rewired contacts (fig. 6). In contrast, conserved sites reveal a monotonic increase in $f_{r}$, such as, at sequence distances of approximately 70%, changes in their contact shells are as large as the expected changes in $f_{r}$ at substituted sites (fig. 6). In other words, at sequence divergences of >70%, the effect of genetic background on the contact shell of conserved sites vanishes, becoming undistinguishable from the contact shell of substituted residues.

## Discussion

A currently debated question is whether the SSAP of protein homologs is conserved, or depends on sequence divergence (Pollock and Goldstein 2014). Here, we developed a computational procedure to estimate the SSAP of a protein based on structural information and the effect of point mutations on thermodynamic stability. Predictions were able to largely recapitulate the SSAP reported in a mutagenesis experiment of the GB1 protein, suggesting that our approach is relatively accurate and that thermodynamic stability can substantially contribute to the SSAP of proteins. We used our computational procedure to study a large sample of diverse homologous structure pairs, and showed that the contribution of thermodynamic stability alone can lead to a monotonic increase of up to 25–30% of significant differences between SSAP, as a function of sequence divergence. Our conservative analysis revealed that differences in SSAP are consistent across a structurally diverse set of homologous proteins; and identified structural rearrangements as an important force driving differences in SSAP.

Thermodynamic stability is a universal biophysical force known to impact the viability of proteins in the intracellular media, and is therefore, a strong determinant of protein evolutionary rate (DePristo et al. 2005; Tokuriki and Tawfik 2009). Thermodynamic stability might be strongly promoted under environmental conditions, such as high temperatures (Sterner and Liebl 2001); or in populations of large size (Goldstein 2011). Under such conditions, the relation between stability and fitness is well explained by a model of maximum stability (Goldstein 2011). However, thermodynamic stability might not necessarily be under strong selection. In fact, several authors have reported cases in which a protein seems only marginally stable, suggesting the existence of a critical level of stability up to which proteins can afford to remain folded (i.e., threshold stability model). Marginal stability might be simply the result of genetic drift (Goldstein 2011); or the effect of purifying selection on conflicting molecular traits such as function and flexibility (Arnold et al. 2001; Tokuriki et al. 2008). Notably, our work shows that under alternative biophysical models simulating these regimes of selection for stability, the contribution of thermodynamic stability is sufficient to induce significant changes in the SSAP of divergent homologous proteins.

The case has been made that, due to the conservation of a protein's structure and function, amino acid substitutions at equivalent sites should preserve changes in the thermodynamic stability of closely, as well as distant homologous proteins (Ashenberg et al. 2013; Risso et al. 2014; Doud et al. 2015). Indeed, our results support this observation by showing that regardless of sequence divergence, a large fraction of sites shows relatively similar effects on thermodynamic stability. However, our results also revealed that a significant fraction of equivalent sites, as large as ∼30%, can be strongly impacted by sequence divergence (i.e., genetic background).

Why does selection for thermodynamic stability lead to significant changes in the SSAP of divergent homologous proteins? A well-known result from classic comparative studies of protein structures is that amino acid substitutions between homologs can lead to the exponential accumulation of structural deviations (Chothia and Lesk 1986). These deviations are most likely the result of amino acid substitutions at buried sites, which in turn are more likely to contribute to changes in thermodynamic stability, and at sequence distances of 70%, are predicted to induce deviations larger than 2.0 Å (Chothia and Lesk 1986). Indeed, structural analyses of pairs of homologous structures revealed that regardless of sequence divergence, sites with amino acid substitutions rewire on average 30–40% of their surrounding contacts. Furthermore, most sites with significant differences in SSAP are buried and substituted by bulkier amino acids, and most sites responsible for the conservation of stability should be at buried positions. Overall, our observations support recent comparative analyses on the influence of contact density on protein evolutionary rate (Marcos and Echave 2015); and suggest that the rewiring of residue contacts due to structural deviations between homologs is an important determinant of differences in the amino acid preference of proteins.

Our work suffers from several limitations. On the one hand, our predictions rely extensively on the accuracy of force fields for the estimation of thermodynamic stability. Indeed, calculations performed by FoldX only optimize the atomic coordinates of amino acid side chains, while leaving backbone atoms constant. It has been shown, however, that methods such as Rosetta or Modeller, which account for variation in both side chains and backbone atoms, do not perform better (Kellogg et al. 2011). In addition, methods that could potentially lead to better predictions are often slow and computationally costly, making a large-scale analysis like ours, unfeasible. Consequently, we used the force field implemented in FoldX and sought to minimize the influence of factors that affect predictions of thermodynamic stability by focusing on a representative sample of high-resolution, single-domain crystal structures, with no cocrystalized ligands, modified, or incomplete residues. Although computational predictions do not need to fully capture deviations in thermodynamic stability to be informative about the overall effect of sequence divergence in the SSAP of proteins, the prediction of multiple mutations per site improves the accuracy of force fields (Capriotti et al. 2008; Tian et al. 2010). Similarly, mutations of large-effect are more likely to be correctly predicted, suggesting that SSAP with nonuniform distributions can counteract the effect of wrongly predicted thermodynamic stability at a given site. Consequently, our computational pipeline was able to recapitulate to a large extent an SSAP profile obtained experimentally (Olson et al. 2014).

Another limitation of our predictions relates to the use of the JS distance for the measurement of differences between SSAP. This metric does not account for the fact that some amino acid substitutions would be more prone to preserve the physicochemical properties of a site. Thus, our predictions might be overestimating changes in the magnitude of amino acids with similar properties, and underestimating smaller deviations toward amino acids with strong differences in their physicochemical properties. Although the goal of the JS distance is not to directly assess the effect of SSAP on the entrenchment of mutations, deviations in the conservation of physicochemical properties might have a large impact in assessing the performance of experimentally derived substitution models for phylogenetics.

Overall, our analyses revealed that sequence divergences of up to 40% translate into 10–15% significant differences in SSAP, which is in relative agreement with experimental studies reporting differences in SSAP of the order of 3–15% between closely related homologs (Doud et al. 2015; Chan et al. 2017). In addition, our analyses suggest that divergent homologs (i.e., sequence distances >70%) can reach up to 25–30% of sites with significantly different SSAP. Our observations find support in simulation studies demonstrating the existence of strong epistatic effects between mutations (Pollock et al. 2012; Shah et al. 2015; Starr and Thornton 2016). Similarly, a recent experimental study that reconstructed the deep evolutionary history of the N-terminal domain of HSP90 found a large fraction of epistatic interactions (Starr et al. 2018). In particular, the study showed that more than 80% of all amino acid substitutions in HSP90 ancestral sequences, spanning up to 30% in divergence, are deleterious in the genetic background of the extant HSP90 sequence of *Saccharomycescerevisiae*. These findings suggest that both epistasis as well as differences at SSAP should be fairly common (Starr et al. 2018). In this regard, we note that our analyses were conservative, and that although we only focused on thermodynamic stability, several other factors might contribute to differences between the SSAP of homologs, such as, selection for function, or insertions and deletions at nonequivalent sites. For instance, at least 5% of the significant differences between observed and predicted profiles of the GB1 protein are due to mutations at sites involved in GB1’s molecular function (figs. 2*C* and 3). Similarly, several epistatic substitutions in the HSP90 experiment described above were shown to be due to functional constraints, as well as interactions between residues of different structural domains of the HSP90 (Starr et al. 2018).

Even though close homologs can have significant differences in SSAP (∼10–20%), experimentally derived substitution models might still be able to substantially outperform the phylogenetic fit of traditional models of amino acid substitution (Bloom 2014). As reported previously, however, even differences in SSAP of the order of 3–15%, can have a detectable impact on the use of experimentally derived substitution models for phylogenetics (Doud et al. 2015). Consequently, our results suggest that, the use of this type of models for phylogenetic analyses of largely divergent homologs might be subject to significant, unforeseen deviations. Future work should explore the extent and consequences of the variation in preference profiles for the accurate estimation of phylogenetic trees and other applications, as well as more sophisticated models that integrate both the biophysics and evolutionary aspects of amino acid substitutions in proteins.

## Supplementary Material

Supplementary data are available at *Genome Biology and Evolution* online.

## Supplementary Material

Supplementary Data
