# Supplementary material for: The Site-Specific Amino Acid Preferences of Homologous Proteins Depend on Sequence Divergence
Source: Genome Biol Evol. 2018 Nov 29;11(1):121–35. doi: 10.1093/gbe/evy261 (PMC6326188; doi:10.1093/gbe/evy261)
Supplement: Supplementary Data [file evy261_supp.pdf]

Supplementary Material

The site-specific amino acid preferences of homologous proteins depend on sequence divergence

Evandro Ferrada*[1]

[1] Centro de Genómica y Bioinformática, Universidad Mayor. Camino La Pirámide 5750, Huechuraba, Santiago 8580745, Chile.

*email: evandro.ferrada@mayor.cl

Supplementary text

Fitness models based on protein biophysics

As described in the main text, we calculate SSAP using a biophysical model of protein folding, which provides a mapping between mutation and fitness. We follow previous works (Pollock et al., 2012; Echave et al., 2015; Doud et al., 2015); and define the propensity of a site r to be occupied by amino acid a, as:

$$\pi_{r,a} = \frac{P_f(a)}{\sum_{j\in\mathcal{A}} P_f(j)} \tag{1}$$

with $\mathcal{A}$, the set of 20 proteinaceous amino acids. SSAP for site r are summarized by the 20-component vector: $\vec{\pi}_r$. The full SSAP profile, with 20xL entries, with L, the protein length; is represented by the matrix $\boldsymbol{\pi} = [\vec{\pi}_1, ..., \vec{\pi}_L]$.

A biophysical model provides a quantitative relation between changes in thermodynamic stability caused by mutation, and a protein's propensity to fold, or folding probability (P_f). Considerable empirical evidence exists that relates misfolding, protein aggregation and toxicity, which translates into organismal fitness (Drummond and Wilke, 2008; Soskine and Tawfik, 2010). There have been 3 main alternative models proposed to explain the effects of protein biophysics on fitness (Echave and Wilke, 2017; Bershtein et al., 2017).

The threshold stability model. This model is based on Boltzmann statistics (Dill and Bromberg, 2003); and to the best of my knowledge it has been derived in the context of protein fitness, independently, several times (Tokuriki and Tawfik, 2009; Wylie and Shakhnovich, 2011; Goldstein 2011). According to the model, the folding probability of a protein can be calculated from the protein's ΔG, as:

$$P_f(a) = \frac{1}{1 + e^{\Delta G_{r,a}/kT}} = \frac{e^{-\Delta G_{r,a}/kT}}{1 + e^{-\Delta G_{r,a}/kT}} \tag{2}$$

In our analyses ΔG, corresponds to the Gibbs free energy associated to a single mutant variant at site r, and its relation to the stability of the reference sequence (wt), can be obtained as:

$$\Delta\Delta G = \Delta G_{r,a} - \Delta G_{r,wt} \tag{3}$$

Substituting $\Delta G_{r,a}$ in Eq. (2):

$$P_f(a) = \frac{e^{-(\Delta\Delta G_{r,a} + \Delta G_{r,wt})/kT}}{1 + e^{-(\Delta\Delta G_{r,a} + \Delta G_{r,wt})/kT}} \tag{4}$$

This model can be represented by a sigmoidal function centered at $\Delta G_{r,wt}$. Therefore, mutations causing negative deviations from $\Delta G_{r,wt}$ ($\Delta\Delta G < 0$), are either beneficial or neutral, while positive

deviations reduce fitness. By approximating $\Delta G_{r,wt} \approx 0$, Eq (4) becomes:

$$P_f(a) = \frac{e^{-\Delta\Delta G_{r,a}/kT}}{1 + e^{-\Delta\Delta G_{r,a}/kT}} \tag{5}$$

A justification for using Eq (5) is that structural data often harbor substantial error, which can be measured as a the resolution of the protein's X-ray crystal. This error has a direct impact on calculations of thermodynamic stability, which are carried out on the optimized input structure. Indeed, we observe a strong association between the resolution of crystal structures and thermodynamic stability in our control dataset (Pearson correlation r = 0.77; p-value=3.67E-73) (Figure S0Y). This observation suggests that error in structural data might strongly influence the expected ground thermodynamic stability in natural proteins, which often ranges between -15 to -5 kcal/mol (Fersht et al., 1999). In order to remove this source of error one might assume that $\Delta G_{r,wt} \approx 0$, under the main assumption that structural error is randomly distributed throughout the crystal, that is, no single mutation can drastically optimize the structure's quality. Restricting our analyses to structural data of high resolution shows that the approximation introduced in Eq (5) does not affect our conclusions (Figure S3). See also (Tokuriki and Tawfik, 2009; Echave et al. 2015; Shah et al., 2015),

The maximum stability model. The maximum stability model, assumes that stabilizing mutations translate proportionally into a larger folding probability (Bloom et al., 2005; Echave et al., 2015; Echave and Wilke, 2017). The model can be generally expressed as:

$$P_f(a) = \alpha \cdot exp[-\lambda \cdot (\Delta\Delta G_{r,a})^n] \tag{6}$$

With n=1 and $\lambda = 1/kT$ (Bloom et al., 2005; Echave et al., 2005; Echave and Wilke, 2017). The constant α vanishes in Eq (1). Echave et al. (2015), derived the maximum stability model and used structural data to fit the parameter λ. They showed that under the normalization in Eq. (1), one can safely assume $\lambda = 1$ (see Eq. 13 and Figure 1 in Echave et al. (2015)). For further details on the derivation of the maximum stability model see Echave et al., (2015).

The optimum stability model. Using n=2 in Eq (6), we obtain the optimum stability model (DePristo et al., 2005; Goldstein 2011; Shah et al., 2015; Echave and Wilke, 2017). According to this model, P_f is described as a Gaussian distribution, where protein function is optimal at the thermodynamic stability of the reference sequence, and both positive and negative deviations in stability, reduce P_f. As in the case of the maximum stability model, the constant α vanishes in Eq (1). As in the analysis by Echave et al., (2015), λ should be fitted to empirical data and is likely idiosyncratic to specific proteins. However, as observed in the case of the maximum stability model, normalization should reduce the impact of variation in λ (Echave et al., 2015). Depristo et al., (2005) adopted λ ranges between 0.3 and 0.5. We set $\lambda = 1.0$.

Protein sites involved in GB1 molecular function explain unexpected deviations in amino acid preferences

Here we provide additional evidence that supports the observation that strong deviations in observed versus predicted SSAP are associated to biophysical determinants other than thermodynamic stability.

Site 22 might play a role in GB1 folding kinetics. Most predicted SSAP with strong, significant deviations with respect to the observed preference profile, have similarly uniform distributions of amino acid preferences (*i.e.* 27,31,35; Figure 2C). In contrast to this pattern, site 22 is not part of the binding interface, and is predicted to deviate significantly to an Asp or Glu residues (P-value <0.01), while its observed preference profile shows an almost uniform SSAP (Figure 2C). Our prediction of a biased Asp/Glu site 22 is in agreement with previous studies showing that an acidic side chain (*i.e.* Asp/Glu) can stabilize residue 25 (Gronenborn et al., 1991); a highly favorable interaction for GB1's overall stability (Malakauskas and Mayo, 1998). Interestingly, however, an *in vitro* study demonstrated that residue 22 also plays a role on GB1's folding kinetics (McCallister et al., 2000), an effect that can not be captured by our predictions, and might be responsible for the more uniform SSAP observed at position 22 in the experimental data.

Spatial proximity to functional sites. An additional insight from the analysis of GB1's crystal structure is that 3 of the sites with significant deviations in their amino acid preferences (*i.e.* 5, 45, 54); are in close proximity to residues involved directly in the GB1 binding interface. In particular, residue 43 seems to interact directly with residues 5 and 54; while residue 45 with residue 27 (residues in blue, Figure 3). In order to provide a quantitative analysis of the relation of SSAP at nearby positions to GB1 binding interface, we constructed a residue contact network by averaging residue interactions across all 1,064 single mutant models of GB1 used to derive the predicted profile in Figure 2C (Methods). Thus, if *in silico* was to perfectly capture *in vitro* mutagenesis, edges between residues in this network would represent the fraction of times that two residues interacted across all variants assayed in the mutagenesis experiment.

The residue contact network reveals strongly connected groups of residues, such as 30–5–7–54, that engage in multiple direct and indirect contacts with residues 31, 43 and 27, suggesting that they might be impacted by GB1's binding function (Figure S2). To test this hypothesis, we study the minimum distance in number of edges (*i.e.* shortest path length), necessary to connect a residue to any of the 5 sites directly involved in GB1's binding function (*i.e.* 27, 28, 31, 35 and 43). We observe that the remaining 4 sites with significant differences in SSAP (*i.e.* 5, 22, 45, 54) are on average at a shortest path length of 3 steps away from any of the residues in the GB1's binding interface. In order to assess whether an average short path length of size 3 is statistically closer to the binding interface of GB1, we constructed a null distribution by calculating the shortest path length between 4 randomly selected sites from the whole network, to any of the residues directly involved

in the binding interface (Methods). As hypothesized, the remaining 4 significantly different sites (*i.e.* 5, 22, 45, 54), are on average closer than expected to residues at the GB1's binding interface (P-value = 2.4x10^{-6}; one-sided, exact Wilcoxon Rank Sum test).

Code availability

`https://github.com/eferrada/GBE_SSAPs`

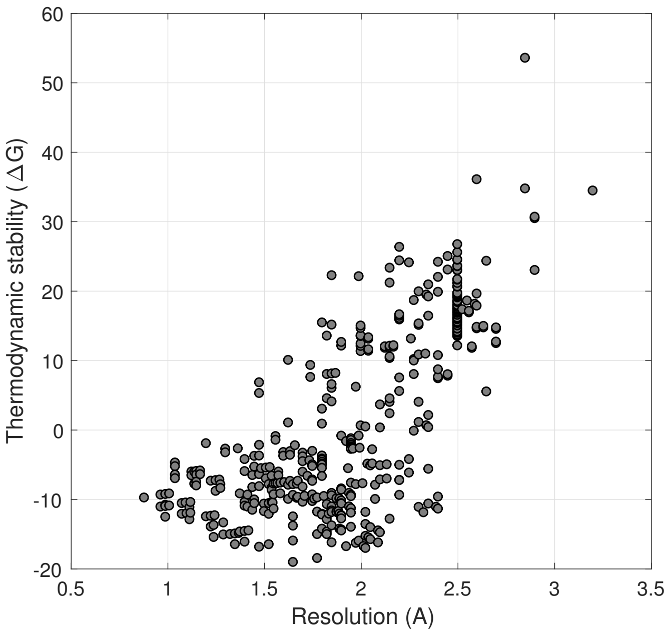

Figure S1. **Significant positive association between the thermodynamic stability and the quality of protein structural data**. Sample of 426 pairwise comparisons of the immunoglobulin-binding fold. Each point summarize the average resolution versus the average thermodynamic stability of a pair of structures. Pearson correlation coefficient r=0.77, P-value=3.67x10^{-73}.

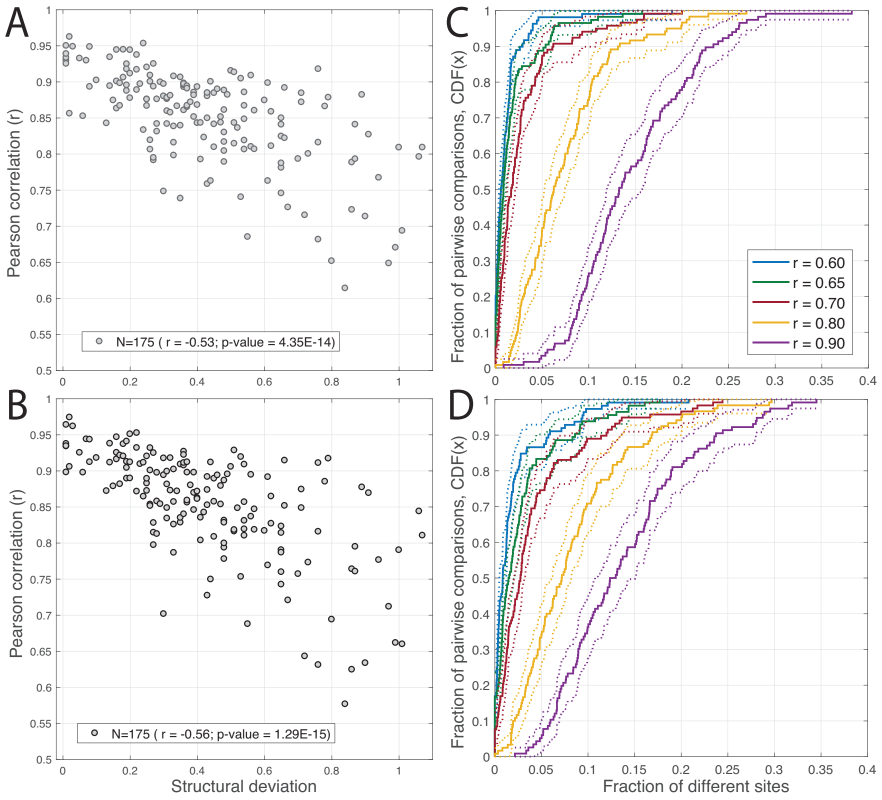

Figure S2. **Alternative biophysical models and the effect of structural deviations on the prediction of amino acid preferences**. (A,B) Association between structural deviations and the Pearson correlation coefficient for a set of 175 structure pairs with 100% sequence identity using the optimum (A), and the maximum (B) stability models. Structural deviations were measured as the residual root-mean square error between C_α carbons. Correlations between preference profiles were calculated according to the Pearson coefficient. (C,D) Cumulative distribution of the fraction of different sites per pairwise comparison versus the fraction of comparisons in the set of 175 structure pairs of identical sequences using the optimum (C), and the maximum (D) stability models. Comparisons of SSAP profiles were carried out using Doud et al.'s method at an α of 0.01. For each structure in the comparison, a pair of replicate profiles were simulated with Pearson correlation coefficients of 0.9 (purple), 0.8 (yellow), and 0.7 (red), 0.65 (green), and 0.60 (blue).

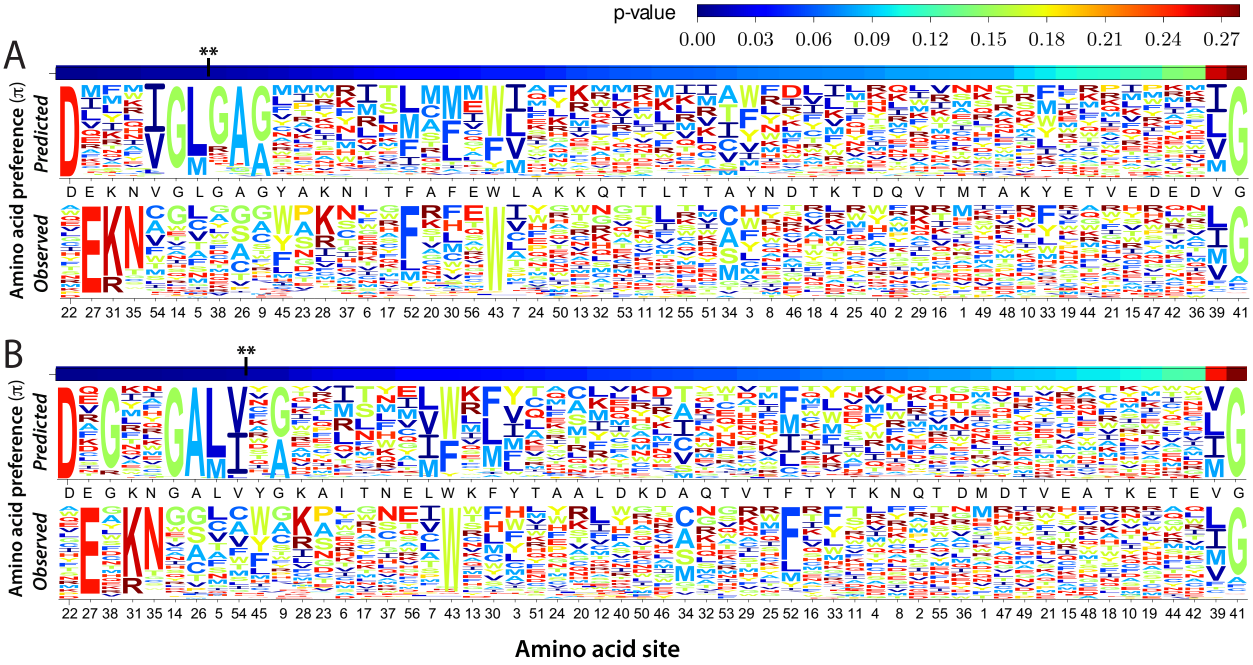

Figure S3. **Predicted preference profiles for GB1 protein using alternative biophysical models for the effect of thermodynamic stability on fitness**. GB1 preference profiles were predicted using GB1 crystal structure (PDB:2gi9), and the pipeline described in Figure 1. Predicted profile of GB1 protein using the threshold (**A**) and the optimum (**B**) stability models. Sequence logos were constructed using the routine *dms_plotlogo* (Bloom, 2015). Sites were sorted according to their *P*-value (**, *P*-value = 0.01).

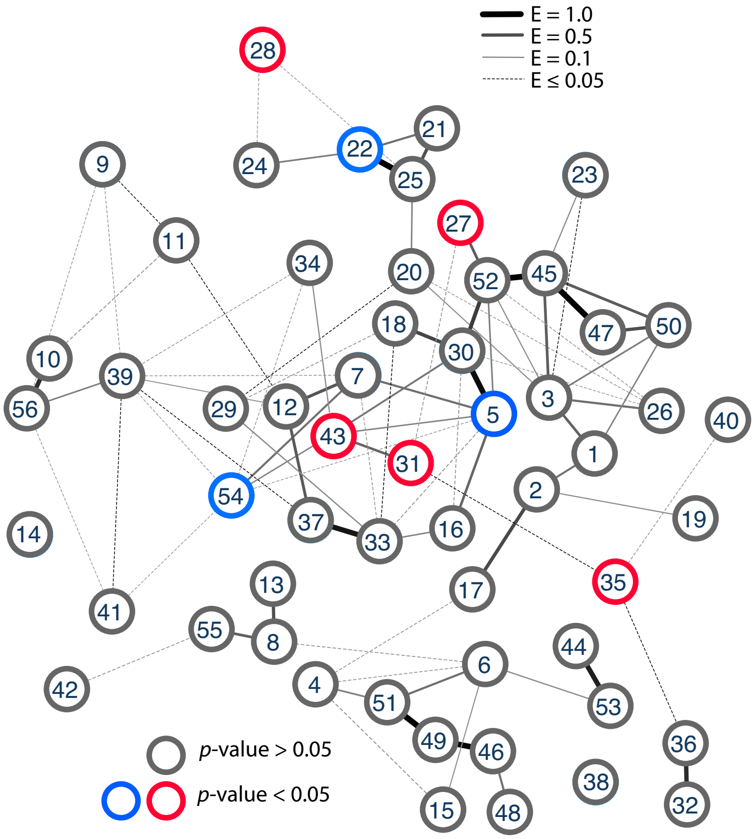

Figure S4. **Residue contact network derived from all single-mutant models of the GB1 protein.** A residue contact network was constructed by calculating the fraction of times (E) any of the side-chain atoms of a pair of residues were observed at <3.5Å, across all 1,064 single-mutant structure models of GB1. Edges were classified according to the frequency of interaction between the pair of sites (E). Nodes were colored according to *P*-values obtained by comparing GB1 predicted versus observed preference profiles through the Doud et al. method (Methods). Nodes with significant differences in SSAP where classified as directly (red), or indirectly (blue) involved in the GB1 binding interface (cf. Figure 3).

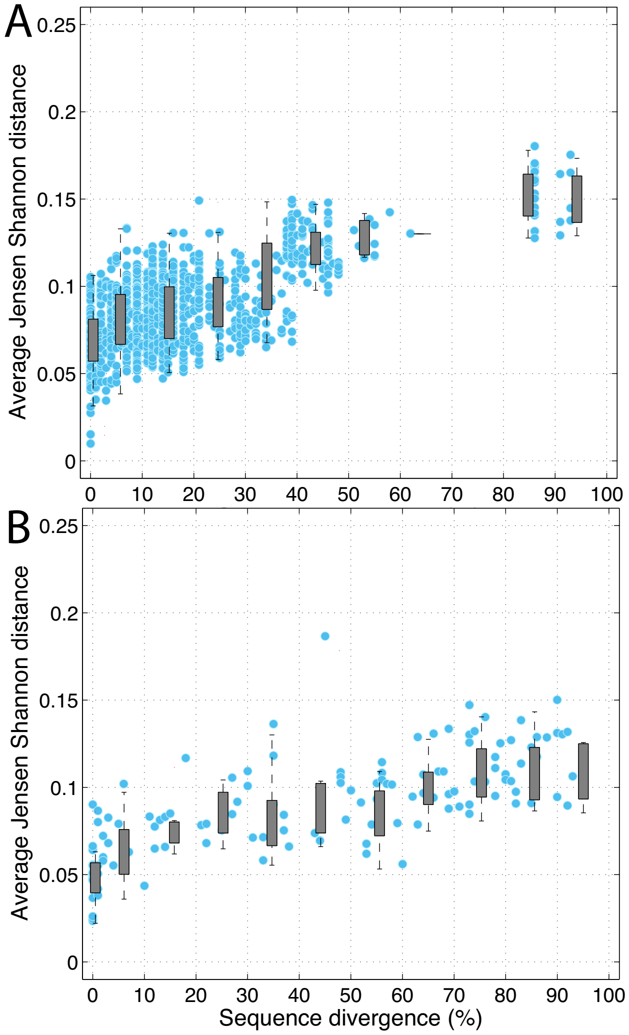

Figure S5. **Average Jensen-Shannon distance versus sequence divergence.** Average JS distance between pairs of SSAP profiles as a function of the sequence divergence. The JS distance is the squared root of the JS divergence (Lin 1989). (**A**). Immunoglobulin-binding family. Pairwise comparisons of sequences resulted in 870 significant alignments. (**B**). Set of 124 representative families (Methods).

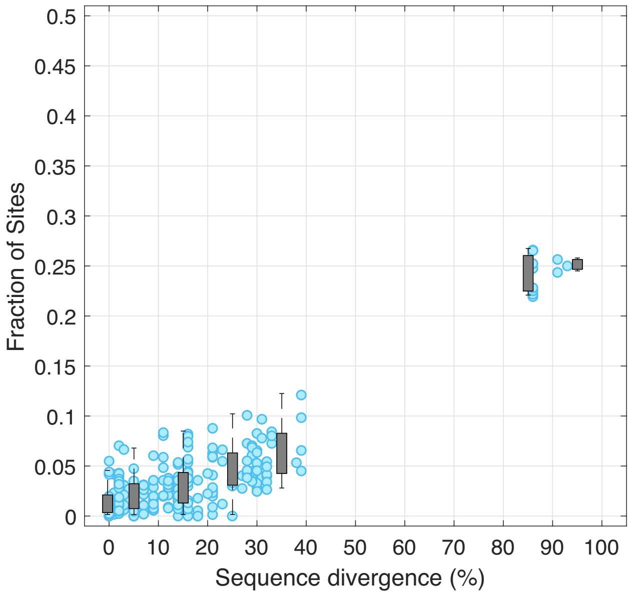

Figure S6. **The site-specific amino acid preferences of homologous proteins depends on sequence divergence in a set of homologs with high-quality structures.** Fraction of sites with significant differences in SSAP (P-value<0.01), as a function of the sequence divergence between pairs of Ig-binding homologs. We selected a subset of 40 Ig-binding domain homologs with resolutions better than 2.5 Å, or solved by NMR but with conservative initial thermodynamic stabilities.

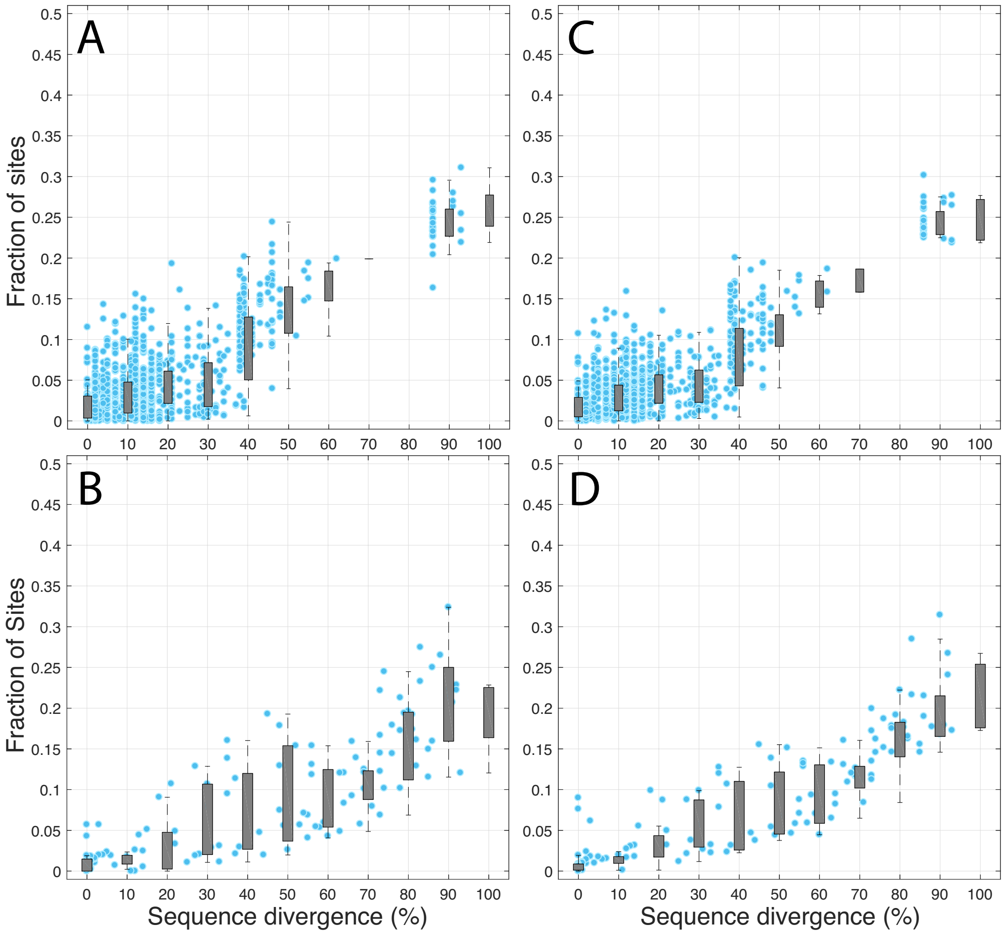

Figure S7. **The site-specific amino acid preferences of homologous proteins depend on sequence divergence under alternative models for the effect of thermodynamic stability on fitness.** Fraction of sites with significant differences in SSAP (P-value<0.01), as a function of the sequence divergence between pairs of homologous structures of the Ig-binding family (**A**, **C**); and a representative set of SCOP families (**B**, **D**). The same two sets of homologs compared in Figure 4 were compared using the threshold stability model (**A**, **B**); and the optimum stability model (**C**, **D**). For details, see Methods and legend of Figure 4.

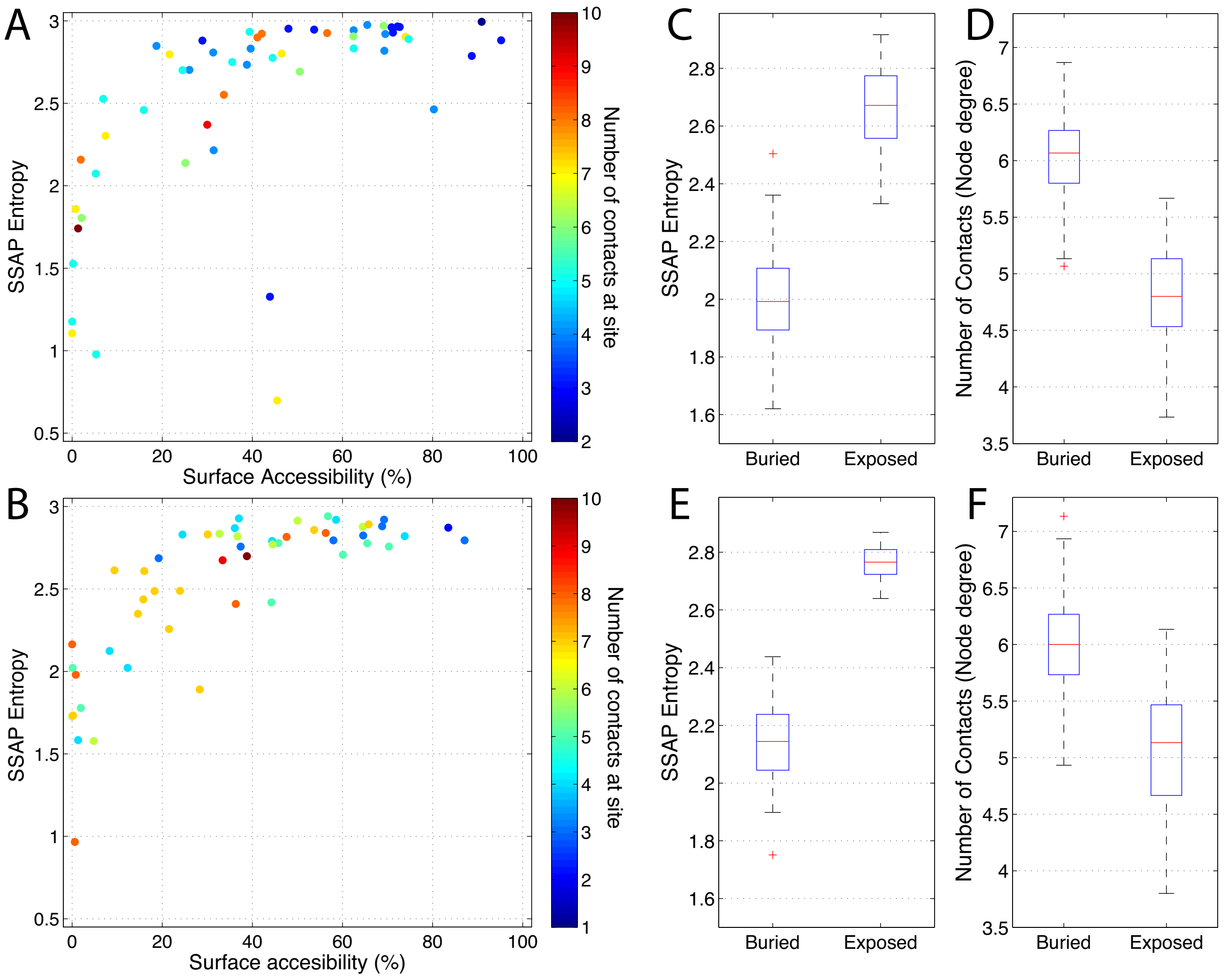

Figure S8. **Exposed sites have more uniform SSAP and interact with fewer other sites.** The uniformity of a SSAP was estimated by the SSAP Entropy (S), as: $S(i) = \sum_{j\in\mathcal{A}} \pi_i(j)\ ln[\pi_i(j)]$. With π_i, the SSAP at site i; and $\mathcal{A}$, the amino acid alphabet. Two residues were defined in physical contact, if any of their atoms were at an Euclidean distance lower than 3.5Å. Sites were defined as exposed or buried at a surface accessibility of 25%. (**A,B**). Entropy per SSAP versus surface accessibility for the 1uwx (**A**); and 1hez (**B**) homologs. (**C-F**). Differences in SSAP Entropy and number of contacts between buried and exposed sites in the 1uwx (**C,D**); and 1heze (**E,F**) homologs. Only 15 (1uwx) and 20 (1hez) sites were found to be buried. Thus, we compared buried versus exposed sites by sampling either 15 or 20 sites from the buried and exposed categories, with replacement, 10,000 times. We estimated a P-value by calculating the fraction of times that the median of the sampled distributions of exposed sites were smaller than the median of the distribution of buried sites. We found: (**C**) P-value = 0.0001; (**D**) P-value = 0.802 (n.s); (**E**) P-value = 0 ; (**F**) P-value = 0.913 (n.s).

Tables

Table S1: Representative set of pairs of homologs with 100% sequence identity.

ID1	Length	ID2	Family	Description
d1pd3b_	54	d1pd3a_	a.30.3.1	Nonstructural protein ns2, Nep, M1-binding domain
d1ycyd_	62	d1ycyb_	b.38.1.4	PF1955-like
d2h4ob_	62	d2h4oa1	d.368.1.1	Yonk-like
d2nn4c_	62	d2nn4a1	a.272.1.1	YqgQ-like
d2fklb_	64	d2fkla_	d.230.3.1	Amyloid beta a4 protein copper binding domain (domain 2)
d1k1ff_	66	d1k1fd_	a.147.1.1	Bcr-Abl oncoprotein oligomerization domain
d2axud1	66	d2axuc1	a.35.1.11	PrgX N-terminal domain-like
d4bwsf_	66	d4bwsc_	d.76.1.1	GYF domain
d1no1c_	67	d1no1a_	a.179.1.1	Replisome organizer (g39p helicase loader/inhibitor protein)
d1vp7e_	67	d1vp7a_	a.7.13.1	XseB-like
d2oauf1	67	d2oaue1	b.38.1.3	Mechanosensitive channel protein MscS (YggB), middle domain
d4icgd_	67	d4icgc_	a.23.5.1	Hemolysin expression modulating protein HHA
d1o9yb_	70	d1o9ya_	b.139.1.1	Surface presentation of antigens (SPOA)
d2dlbb_	70	d2dlba1	b.174.1.1	YopT-like
d1k3rb1	71	d1k3ra1	b.40.4.10	Hypothetical protein MTH1 (MT0001), insert domain
d3bzca2	73	d2ocea2	a.60.2.6	Tex HhH-containing domain-like
d1mbyb_	75	d1mbya_	d.223.1.1	Swapped Polo-box domain
d1r0vd3	75	d1r0va3	d.75.1.1	tRNA-intron endonuclease N-terminal domain-like
d1qdmc1	77	d1qdmb1	a.64.1.2	Swaposin
d1qiuc2	77	d1qiub2	b.83.1.1	Adenovirus
d1rp3e1	77	d1rp3c1	a.4.13.1	Sigma3 domain
d2gr8b_	77	d2gr8a1	d.24.1.4	YadA C-terminal domain-like
d2ffgb_	78	d2ffga1	d.317.1.1	YkuJ-like
d2p4vd1	79	d2p4va1	a.2.1.0	automated matches
d1bcoa1	80	d1bcmb1	b.48.1.1	mu transposase, C-terminal domain
d2gbob_	82	d2gboa1	a.23.6.1	EF2458-like
d2fhza1	83	d2dfxi1	d.311.1.1	ImmE5-like
d1wz3b_	84	d1wz3a1	d.15.1.7	APG12-like
d1stzc1	85	d1stzb1	a.4.5.51	Heat-inducible transcription repressor HrcA, N-terminal domain
d1z3ya1	85	d1z3xa1	a.118.1.22	GUN4-associated domain
d1x6ja_	87	d1x6ib_	a.218.1.0	automated matches
d3le5b_	87	d3le5a_	d.94.1.0	automated matches
d2esnb1	89	d2esna1	a.4.5.37	LysR-like transcriptional regulators
d3ncwb1	89	d3ncwa1	b.1.14.0	automated matches
d1l0qc1	90	d1l0qa1	b.1.3.1	PKD domain
d1w2ea_	90	d1t3ud_	d.244.1.1	Cell division protein ZapA-like
d2b26a2	90	d1c3ga2	b.4.1.1	HSP40/DnaJ peptide-binding domain
d2x3de_	90	d2x3dd_	d.58.61.0	automated matches
d1hl8b1	91	d1hl8a1	b.71.1.3	Putative alpha-L-fucosidase C-terminal domain
d3cw2g3	91	d3cw2d3	d.58.51.1	eIF-2-alpha, C-terminal domain
d3k6cd_	91	d3k6ca_	a.25.1.5	half-ferritin
d2dhhc4	92	d2dhhb4	d.225.1.1	Multidrug efflux transporter AcrB TolC docking domain; DN and DC subdomains
d2fdob_	92	d2fdoa1	d.337.1.1	AF2331-like
d2v94b1	92	d2v94a1	d.12.1.3	Ribosomal protein S24e
d1t5ya2	94	d1sqwa2	d.17.6.3	Nip7p homolog, N-terminal domain
d1yf5l1	94	d1w97l2	c.55.1.11	Cyto-EpsL domain
d2cxdb_	94	d2cwya1	a.246.2.1	TTHA0068-like
d1im3l_	95	d1im3h_	b.1.18.5	Cytomegalovirus protein US2
d1qmid1	95	d1qmib1	c.47.2.1	RNA 3'-terminal phosphate cyclase, RPTC, insert domain
d2fiub_	95	d2fiua1	d.58.4.16	Atu0297-like
d4nftb1	95	d4nfta1	a.22.1.0	automated matches
d1d2pa4	96	d1d2ob2	b.3.5.1	Cna protein B-type domain
d2epid_	96	d2ekyh_	d.58.48.0	automated matches

Table S1 (continue): Representative set of pairs of homologs with 100% sequence identity.

ID1	Length	ID2	Family	Description
d1f02i3	98	d1f00i3	d.169.1.3	Invasin/intimin cell-adhesion fragment, C-terminal domain
d1ykec1	98	d1ykea1	a.252.1.2	MED7 hinge region
d1ka8d_	100	d1ka8a_	a.4.5.20	P4 origin-binding domain-like
d1ylxb_	100	d1ylxa1	d.82.5.1	GK1464-like
d1jnpb_	101	d1jnpa_	b.63.1.1	Oncogene products
d2vv5f2	101	d2oaua2	d.58.43.1	Mechanosensitive channel protein MscS (YggB), C-terminal domain
d3whob_	101	d3whoa_	d.1.1.0	automated matches
d1b79c_	102	d1b79a_	a.81.1.1	N-terminal domain of DnaB helicase
d1qsdb_	102	d1qsda_	a.7.5.1	Tubulin chaperone cofactor A
d2fswb_	102	d2fswa1	a.4.5.69	HxlR-like
d2es7b_	103	d2es7a1	c.47.1.20	HyaE-like
d2gtcc_	103	d2gtca_	d.230.5.1	YbjQ-like
d1lktf_	104	d1lktd_	b.90.1.1	Head-binding domain of phage P22 tailspike protein
d1kptb_	105	d1kpta_	d.70.1.1	Virally encoded KP4 toxin
d1v8hb_	106	d1v8ha1	b.1.18.19	SoxZ-like
d2av5d_	106	d2av5b_	d.58.59.1	Rpp14/Pop5-like
d2c0fa1	106	d2c0ea1	a.71.1.0	automated matches
d1hf2d1	107	d1hf2b1	b.80.3.1	Cell-division inhibitor MinC, C-terminal domain
d1kafc_	108	d1kafb_	d.199.1.1	DNA-binding C-terminal domain of the transcription factor MotA
d2eucb_	108	d2euca1	a.249.1.1	YfmB-like
d2pd2b_	108	d2pd2a_	c.114.1.0	automated matches
d2q4pa_	113	d2a3qa1	a.204.1.2	MazG-like
d2j85b_	115	d2j85a1	d.321.1.1	STIV B116-like
d2ywad1	115	d1wtyc_	a.24.16.2	Family 1 bi-partite nucleotidyltransferase subunit
d3zylb2	116	d3zyla2	a.7.8.0	automated matches
d1pm4c_	117	d1pm4a_	b.135.1.1	Superantigen (mitogen) Ypm
d3gefc_	117	d3gefb_	b.1.16.1	Lamin A/C globular tail domain
d2dmaa_	118	d2dm9b_	d.81.4.1	V-type ATPase subunit E
d3a4ia2	119	d2dpla2	d.52.2.0	automated matches
d2iojb_	120	d2ioja1	c.98.2.2	DRTGG domain
d2q48a_	120	d1xy7a_	d.32.1.9	Hypothetical protein At5g48480
d2gtdd1	121	d2gtdc1	c.55.1.13	CoaX-like
d2dstb_	122	d2dsta1	c.69.1.39	TTHA1544-like
d2i15b_	122	d2i15a1	a.291.1.1	MG296-like
d4ljxb_	122	d4ljxa_	a.4.3.1	ARID domain
d2pihb_	123	d2piha1	a.281.1.1	YmcA-like
d1orjd_	125	d1orja_	a.24.19.1	Flagellar export chaperone FliS
d2gwfd_	125	d2fzpa1	d.345.1.1	USP8 interacting domain
d1ufbd_	127	d1ufbb_	a.24.16.3	HEPN domain
d2c35c_	128	d2c35a1	a.60.8.2	RNA polymerase II subunit RBP4 (RpoF)
d2ohwb_	128	d2ohwa1	d.79.8.1	YueI-like
d1t7sb_	129	d1t7sa_	a.7.7.1	BAG domain
d1k47f2	135	d1k47e2	d.58.26.4	Phosphomevalonate kinase (PMK)
d2gwfe_	136	d2gwfa_	c.46.1.4	Ubiquitin carboxyl-terminal hydrolase 8, USP8
d1tfea_	141	d1aiph2	d.43.1.1	Elongation factor Ts (EF-Ts), dimerisation domain
d1usvh_	141	d1usvd_	d.83.2.1	Activator of Hsp90 ATPase, Aha1
d2a2lc_	142	d2a2la1	d.110.9.1	GlcG-like
d2gu0b1	142	d2gu0a1	d.216.1.0	automated matches
d2r7ja2	142	d1l9va2	d.216.1.1	Rotavirus NSP2 fragment, N-terminal domain
d2ffsb_	144	d2ffsa1	d.129.3.7	PA1206-like
d2gidj1	146	d2gidd1	d.18.1.4	Guide RNA binding protein gBP
d1em8c_	147	d1em8a_	c.128.1.1	DNA polymerase III chi subunit
d1dvkb_	149	d1dvka_	a.72.1.1	Functional domain of the splicing factor Prp18
d3bzca5	149	d2ocea5	c.55.3.13	Tex RuvX-like domain-like
d1ku9b_	151	d1ku9a_	a.4.5.36	DNA-binding protein Mj223
d1kjnb_	152	d1kjna_	c.115.1.1	Hypothetical protein MTH777 (MT0777)
d2f9zd_	152	d2f9zc1	d.194.1.3	CheD-like

Table S1 (continue): Representative set of pairs of homologs with 100% sequence identity.

ID1	Length	ID2	Family	Description
d1utyb_	153	d1utya_	b.147.1.1	BTV NS2-like ssRNA-binding domain
d1bpoc1	157	d1bpoa1	a.118.1.4	Clathrin heavy-chain linker domain
d1s7mb_	157	d1s7ma_	b.144.1.1	Trimeric adhesin
d1yjgb_	157	d1yjga_	a.24.12.0	automated matches
d1ytlc_	157	d1ytla1	c.31.1.6	ACDE2-like
d1hjrd_	158	d1hjra_	c.55.3.6	RuvC resolvase
d2gu0b2	159	d2gu0a2	d.13.2.0	automated matches
d2nwie_	160	d2nwid_	d.190.1.3	AF1396-like
d1vgge_	161	d1vggd_	d.256.1.1	Ta1353-like
d2o3ab_	161	d2o3aa1	c.116.1.8	AF0751-like
d1mw5b_	162	d1mw5a_	d.259.1.1	Hypothetical protein HI1480
d1vmob_	163	d1vmoa_	b.77.1.1	Vitelline membrane outer protein-I (VMO-I)
d1mg7b1	164	d1mg7a1	d.14.1.6	Early switch protein XOL-1, N-terminal domain
d2ed6j_	170	d2ed6f_	b.170.1.1	WSSV envelope protein-like
d1x9yc2	171	d1x9yb2	d.17.1.4	Staphopain B, prodomain
d2pv3a1	171	d1m5yd1	a.223.1.2	Porin chaperone SurA, peptide-binding domain
d4gjhc_	176	d4gjhb_	b.8.1.0	automated matches
d1zawa1	177	d1zava1	d.58.62.1	Ribosomal protein L10-like
d2hafa1	177	d2aj2a1	d.310.1.1	VC0467-like
d2ff4a2	179	d2feza2	a.118.8.3	BTAD-like
d2zxxf_	183	d2zxxc_	a.4.5.52	DNA replication factor Cdt1
d1kn0c_	184	d1h2ir_	d.50.1.3	The homologous-pairing domain of Rad52 recombinase
d1w26b1	185	d1w26a1	a.223.1.1	TF C-terminus
d1l3be_	186	d1l3bb_	c.66.1.22	Precorrin-6Y methyltransferase (CbiT)
d1wdjc_	186	d1wdja_	c.52.1.27	Hypothetical protein TT1808 (TTHA1514)
d1xm7b_	186	d1xm7a_	d.159.1.8	Hypothetical protein aq_1666
d2pjuc_	186	d2pjua1	c.92.3.1	PrpR receptor domain-like
d1mg7b2	187	d1mg7a2	d.58.26.6	Early switch protein XOL-1
d1r0de_	190	d1r0dd_	a.216.1.1	I/LWEQ domain
d2cwea1	190	d1ulya_	a.4.5.58	Hypothetical protein PH1932
d2gp4b1	190	d2gp4a1	c.8.2.2	IlvD/EDD C-terminal domain-like
d1k3rb2	191	d1k3ra2	c.116.1.2	Hypothetical protein MTH1 (MT0001), dimerisation domain
d1j6rb_	197	d1j6ra_	d.173.1.2	Hypothetical protein TM0269
d1o5hb_	198	d1o5ha_	a.191.1.1	Methenyltetrahydrofolate cyclohydrolase-like
d2fb5b_	201	d2fb5a1	d.320.1.1	YojJ-like
d1a2ob2	207	d1a2oa2	c.40.1.1	Methylesterase CheB, C-terminal domain
d1el6c_	208	d1el6a_	d.182.1.1	Baseplate structural protein gp11
d1xdob2	208	d1xdoa2	d.322.1.2	PPK middle domain-like
d2icub_	210	d2icua_	d.303.1.0	automated matches
d2icwh_	213	d2icwg_	a.202.1.1	Superantigen MAM
d4gyvd_	216	d4gyva_	a.87.1.0	automated matches
d1yf2b1	220	d1yf2a1	d.287.1.2	Type I restriction modification DNA specificity domain
d2pofb_	220	d2pofa1	d.13.1.4	CDH-like
d1vavb_	222	d1vava_	b.29.1.18	Alginate lyase
d2grkb_	225	d2grka_	b.27.1.1	Soluble secreted chemokine inhibitor, VCCI
d3usyb_	226	d3usya_	a.118.14.0	automated matches
d1yxyb_	230	d1yxya1	c.1.2.5	NanE-like
d2f20b_	231	d2f20a1	d.303.1.1	BB1717-like
d2g9ib_	232	d2g9ia1	d.340.1.1	CofE-like
d1m1lb_	236	d1m1la_	d.260.1.1	Suppressor of Fused, N-terminal domain
d1ufof_	237	d1ufod_	c.69.1.27	Hypothetical protein TT1662
d1xm3d_	249	d1xm3b_	c.1.31.1	ThiG-like
d1l5ab2	250	d1l5aa2	c.43.1.2	NRPS condensation domain (amide synthase)

Table S1 (continue): Representative set of pairs of homologs with 100% sequence identity.

ID1	Length	ID2	Family	Description
d2pifb_	252	d2pifa1	d.382.1.1	PSTPO5379-like
d2pr9a1	254	d1bw8a_	b.2.7.1	Second domain of Mu2 adaptin subunit (ap50) of ap2 adaptor
d4en2a_	254	d4emzm_	b.2.7.0	automated matches
d1t70g_	255	d1t70e_	d.159.1.9	DR1281-like
d2i5ib_	261	d2i5ia1	c.6.2.8	YdjC-like
d2p90b_	267	d2p90a1	c.56.8.1	Cgl1923-like
d1k32f2	281	d1k32d2	b.68.7.1	Tricorn protease N-terminal domain
d1zmbb_	282	d1zmba1	c.23.10.7	Putative acetylxylan esterase-like
d1k32b4	291	d1k32a4	c.14.1.2	Tail specific protease, catalytic domain
d1thtb_	293	d1tha_	c.69.1.13	Thioesterases
d1wlgb_	293	d1wlga_	b.152.1.1	Flagellar hook protein flgE
d1l0qd2	300	d1l0qb2	b.69.2.3	YVTN repeat

Table S2: Immunoglobulin-binding family.

SCOP ID*	Chain	PDB ID	Resolution	Stability (kcal/mol)
**d2zw1a_**	A	2zw1	1.60	-19.4545
**d2onqa_**	A	2onq	1.70	-18.725
**d4ioie_**	E	4ioi	1.95	-17.5982
**d1mhhf_**	F	1mhh	2.10	-16.5489
**d1kh0b_**	B	1kh0	1.90	-16.041
**d4hkze_**	E	4hkz	2.08	-16.0159
**d4hjge_**	E	4hjg	2.00	-15.0786
**d2on8a_**	A	2on8	1.35	-14.355
**d1igda_**	A	1igd	1.10	-13.6198
**d1mhhe_**	E	1mhh	2.10	-13.0749
**d1hz6c_**	C	1hz6	1.70	-12.5713
**d3fila_**	A	3fil	0.88	-11.5427
**d1qkza_**	A	1qkz	1.95	-10.7063
**d2qmta_**	A	2qmt	1.05	-10.6927
**d2igda_**	A	2igd	1.10	-10.5316
**d2gi9a_**	A	2gi9	1.14	-10.38
**d1p7ea_**	A	1p7e	-	-10.0186
**d1uwxb_**	B	1uwx	2.20	-9.33785
**d1hz6b_**	B	1hz6	1.70	-8.27275
**d3filb_**	B	3fil	0.88	-8.08296
d1heze_	E	1hez	2.70	-6.28226
**d1k50c_**	C	1k50	1.80	-6.16074
**d1k52a_**	A	1k52	1.80	-5.64862
**d1hz5b_**	B	1hz5	1.80	-5.43261
**d1uwxa_**	A	1uwx	2.20	-5.25349
**d1em7a_**	A	1em7	2.00	-5.07663
**d1pgaa_**	A	1pga	2.07	-4.81264
**d1hz5a_**	A	1hz5	1.80	-4.4531
**d2zw0a_**	A	2zw0	1.40	-4.09059
**d1hz6a_**	A	1hz6	1.70	-3.92839
**d2rmmb_**	B	2rmm	-	-3.92806
**d1k52b_**	B	1k52	1.80	-3.0865
**d3mp9b_**	B	3mp9	1.20	-2.52383
**d1k50a_**	A	1k50	1.80	-2.27073
**d1p7fa_**	A	1p7f	-	-1.61218
**d3mp9a_**	A	3mp9	1.20	-1.45949
**d2oeda_**	A	2oed	-	-0.956629
**d1pgba_**	A	1pgb	1.92	-0.478372
**d1k53b_**	B	1k53	2.10	0.0275906
**d2rmma_**	A	2rmm	-	0.162385
**d2jzpa_**	A	2jzp	-	0.166369
d1k53a_	A	1k53	2.10	0.525847
d2plpa1	A	2plp	-	0.994118
d3gb1a_	A	3gb1	-	2.78316
d1mhxa_	A	1mhx	1.80	2.90104
d1mi0a_	A	1mi0	1.85	6.07177
d3v3xc_	C	3v3x	2.00	10.8592
d1mvka_	A	1mvk	2.50	11.6274
d1mvki_	I	1mvk	2.50	12.5518
d2nmqa_	A	2nmq	-	13.0559
d3v3xb_	B	3v3x	2.00	13.1794
d1k50b_	B	1k50	1.80	13.4117
d3v3xd_	D	3v3x	2.00	13.8778
d1kh0a_	A	1kh0	1.90	14.2195
d1mvkf_	F	1mvk	2.50	15.245
d1mvkd_	D	1mvk	2.50	15.3553
d2kaca_	A	2kac	-	15.4945
d1mvkl_	L	1mvk	2.50	15.6788

Table S2 (continue): Immunoglobulin-binding family.

SCOP ID*	Chain	PDB ID	Resolution	Stability (kcal/mol)
d3v3xa_	A	3v3x	2.00	16.0223
d1mvkc_	C	1mvk	2.50	16.4489
d1mvkb_	B	1mvk	2.50	16.5398
d1ymhe_	E	1ymh	2.60	17.2029
d1k50d_	D	1k50	1.80	17.3794
d1mvkj_	J	1mvk	2.50	18.2909
d1mvkg_	G	1mvk	2.50	18.3807
d1gb1a_	A	1gb1	-	19.1745
d1mvke_	E	1mvk	2.50	20.47
d1k51a_	A	1k51	1.80	21.1915
d1mvkk_	K	1mvk	2.50	22.7906
d2j52a_	A	2j52	-	23.177
d1jmla_	A	1jml	1.90	23.1925
d1mi0b_	B	1mi0	1.85	24.0931
d2igga_	A	2igg	-	25.3333
d1q10b_	B	1q10	-	26.0018
d1q10a_	A	1q10	-	26.2664
d1igca_	A	1igc	2.60	26.6347
d2gb1a_	A	2gb1	-	30.168
d1mvkh_	H	1mvk	2.50	30.5498
d2lhda_	A	2lhd	-	32.9482
d2j53a_	A	2j53	-	33.6223
d1fccd_	D	1fcc	3.20	34.1824
d2luma_	A	2lum	-	34.4682
d1fccc_	C	1fcc	3.20	34.6045
d2lhea_	A	2lhe	-	37.1165
d1fd6a_	A	1fd6	-	39.1315
d2igha_	A	2igh	-	39.476
d1gb4a_	A	1gb4	-	42.9998
d1mpeb_	B	1mpe	-	46.3146
d1fcla_	A	1fcl	-	46.9831
d1mpec_	C	1mpe	-	47.2915
d1mpea_	A	1mpe	-	48.8783
d1ynte_	E	1ynt	3.10	52.1852
d1ymhf_	F	1ymh	2.60	54.827
d1mped_	D	1mpe	-	55.1749
d1zxha_	A	1zxh	-	91.1784

* Bold SCOP IDs are the high-quality set of structures for the analysis in Fig. S3.

Table S3: Representative SCOP families.

ID1	ID2	Length*	E_r**	Seq Id (%)	Family id	Description
d1u8sb2	d1u8sb1	83	2.12	7	d.58.18.5	Glycine cleavage system transcriptional repressor
d3p91a2	d2xura3	116	2.48	8	d.131.1.0	automated matches
d4e18a2	d1l7ca2	114	2.70	8	a.24.9.1	alpha-catenin/vinculin
d3nflc_	d1n7fb_	85	2.93	9	b.36.1.1	PDZ domain
d1thxa_	d1erta_	105	2.87	10	c.47.1.1	Thioltransferase
d1vfqa_	d1hqz7_	128	2.50	10	d.109.1.2	Cofilin-like
d2zaya_	d2qv0a_	118	2.30	10	c.23.1.0	automated matches
d1wmhb_	d1oeya_	78	2.31	12	d.15.2.2	PB1 domain
d1vhka1	d1nxza1	69	1.55	14	b.122.1.2	YggJ N-terminal domain-like
d2yhfi_	d2c6ua_	118	2.58	14	d.169.1.0	automated matches
d2a61b_	d1lnwg_	130	3.82	15	a.4.5.28	MarR-like transcriptional regulators
d4damk_	d3tqya_	107	2.55	15	b.40.4.0	automated matches
d3dgsb1	d2x9aa_	58	2.31	17	b.37.1.0	automated matches
d3g1gb_	d2eiaa1	71	2.10	17	a.28.3.1	Retrovirus capsid protein C-terminal domain
d2a6ba1	d1tyha_	211	2.45	18	a.132.1.3	TENA/THI-4
d2vgxa1	d2q7fa1	62	1.62	18	a.118.8.1	Tetratricopeptide repeat (TPR)
d1qqgb2	d1p5ta_	102	1.75	19	b.55.1.2	Phosphotyrosine-binding domain (PTB)
d1ttwa_	d1l2wb_	115	2.69	19	d.198.1.1	Type III secretory system chaperone
d1yd8g1	d1wrda1	90	2.62	20	a.7.8.1	GAT domain
d3lfhc_	d3bedb_	126	2.20	20	c.54.1.0	automated matches
d2hxxb_	d2cx6a1	80	2.44	21	c.9.1.1	Barstar-related
d1to0d_	d1o6da_	143	2.70	22	c.116.1.3	YbeA-like
d2csua2	d1oi7a2	141	2.66	22	c.23.4.1	Succinyl-CoA synthetase domains
d3e4ub_	d1buoa_	118	2.26	22	d.42.1.1	BTB/POZ domain
d1mkha_	d1gd7c_	104	2.65	24	b.40.4.4	Myf domain
d1wdva_	d1dbxa_	147	1.78	24	d.116.1.1	YbaK/ProRS associated domain
d1qc6b_	d1ddwa_	105	1.91	26	b.55.1.4	Enabled/VASP homology 1 domain (EVH1 domain)
d3cdda1	d1wrua1	145	2.61	26	b.106.1.1	Baseplate protein-like
d1bq7d_	d1beda_	176	2.61	27	c.47.1.13	DsbA-like
d2ge8a1	d2cjrg_	107	2.15	27	d.254.1.2	Coronavirus nucleocapsid protein
d3buac_	d3bqoa_	192	2.58	27	a.146.1.1	Telomeric repeat binding factor (TRF) dimerisation domain
d3eq5a_	d1l8rb_	97	1.91	27	a.6.1.4	Dachshund-homology domain
d3hg0a1	d2bsdc1	102	2.15	27	b.21.1.3	Lactophage receptor-binding protein head domain
d2j4bd_	d2j49a1	129	1.79	29	d.379.1.1	Taf5 N-terminal domain-like
d2ptha_	d1ryna_	185	1.87	30	c.56.3.1	Peptidyl-tRNA hydrolase-like
d3e7dc_	d1ou0b_	192	1.95	31	c.23.17.1	Precorrin-8X methylmutase CbiC/CobH
d3erjb_	d1q7sb_	110	2.29	31	c.131.1.1	Peptidyl-tRNA hydrolase II
d4qjnc_	d4n1va_	89	2.55	31	a.55.1.0	automated matches
d1sv0d_	d1sv0b_	78	1.76	32	a.60.1.1	Pointed domain
d3wdfa_	d2zhxe_	212	2.01	33	c.18.1.0	automated matches
d1u5oa_	d1gybd_	119	2.20	34	d.17.4.2	NTF2-like
d4mh4a_	d2bjob_	132	2.26	34	d.227.1.0	automated matches
d1ji6a1	d1ciya1	145	1.44	36	b.18.1.3	delta-Endotoxin, C-terminal domain
d2qk0a_	d2hkqb_	72	1.67	36	b.34.10.1	Cap-Gly domain
d1sppa_	d1sfpa_	108	2.15	37	b.23.1.1	Spermadhesin, CUB domain
d4mcta_	d2icta_	89	2.30	37	a.35.1.3	SinR domain-like
d3h91a_	d1knea_	50	0.96	38	b.34.13.2	Chromo domain
d2yvaa_	d1tk9b_	186	0.89	40	c.80.1.3	mono-SIS domain
d4fdfb_	d2ohdc_	134	0.82	41	d.58.21.0	automated matches
d1rowb_	d1m1sa_	106	1.16	42	b.1.11.2	MSP-like
d1zq7a1	d1vaja1	196	3.96	43	d.309.1.1	AMMECR1-like
d1odec_	d1fnja_	114	1.31	44	d.79.1.2	Chorismate mutase
d3gaxb_	d1roaa_	107	2.03	44	d.17.1.2	Cystatins
d4wyma1	d2wlvb_	143	2.69	44	a.73.1.1	Retrovirus capsid protein, N-terminal core domain
d1ygsa_	d1khud_	180	1.84	45	b.26.1.1	SMAD domain

Table S3 (continue): Representative SCOP families.

ID1	ID2	Length*	E_r**	Seq Id (%)	Family id	Description
d4qr0a_	d4es2a_	80	1.97	45	d.58.58.0	automated matches
d1bkra_	d1bhda_	103	1.06	46	a.40.1.1	Calponin-homology domain, CH-domain
d1nkda_	d1b6qa_	55	4.83	47	a.30.1.1	ROP protein
d2dfaa1	d1v6ta_	241	1.25	47	c.6.2.5	LamB/YcsF-like
d1or2a_	d1nfoa_	130	3.04	48	a.24.1.1	Apolipoprotein
d1u00a2	d1dkxa2	115	1.12	50	b.130.1.1	Heat shock protein 70kD (HSP70), peptide-binding domain
d3bh2a_	d3bgtd_	242	1.12	51	d.347.1.0	automated matches
d1u4fd_	d1u4ea_	194	1.52	52	b.1.18.16	Cytoplasmic domain of inward rectifier potassium channel
d3b77f_	d3b77d_	183	2.68	52	b.55.1.13	BPHL domain
d4lzia1	d2w9pc_	87	2.24	52	d.17.1.0	automated matches
d2gn5a_	d1vqfa_	83	2.18	55	b.40.4.7	Phage ssDNA-binding proteins
d2r4qa1	d2r48a1	101	1.02	56	c.44.2.2	PTS system, Fructose specific IIB subunit-like
d2p0gc_	d2ojla_	79	0.74	57	c.47.1.23	Selenoprotein W-related
d2o2aa1	d2hnga1	124	0.91	62	d.33.1.2	SP1558-like
d1mhqa_	d1juqc_	142	1.19	63	a.118.9.2	VHS domain
d2e1ha1	d2dzca1	176	2.31	63	d.104.1.2	Biotin holoenzyme synthetase
d2odmb_	d2odma_	79	2.36	65	a.23.6.0	automated matches
d3v4hb_	d3v4ha_	129	2.29	65	b.157.1.0	automated matches
d4phxc_	d4phxb_	130	2.36	65	b.2.3.0	automated matches
d1etva_	d1etkb_	75	2.10	67	a.4.1.12	FIS-like
d2hjdb_	d1upga_	89	0.81	67	a.2.13.1	Transcriptional repressor TraM
d3o9qa_	d3d6rb1	116	1.43	69	d.299.1.1	Ns1 effector domain-like
d1xs0b_	d1xs0a_	125	2.25	70	d.233.1.1	Inhibitor of vertebrate lysozyme, Ivy
d3p9ha_	d3obsa_	139	2.21	70	d.20.1.2	UEV domain
d1p4ua_	d1na8a_	145	1.34	72	b.1.10.2	gamma-adaptin C-terminal appendage domain-like
d1xi8a1	d1wu2b1	70	0.78	73	b.85.6.1	MoeA C-terminal domain-like
d3b48d_	d3b48a1	127	2.01	73	c.54.1.2	DhaM-like
d1wkob_	d1qoub_	156	0.91	75	b.17.1.1	Phosphatidylethanolamine binding protein
d2gt2d_	d2gt2b_	150	1.80	78	d.113.1.5	GDP-mannose mannosyl hydrolase NudD
d4j7bb2	d1umwa2	90	1.14	78	d.223.1.2	Polo-box duplicated region
d2gmqb_	d2gmqa1	98	1.78	79	b.122.1.11	PrgU-like
d2ii8c_	d2ii7f_	91	1.51	82	b.123.1.0	automated matches
d2i1sb_	d2i1sa1	176	1.57	85	d.343.1.1	MM3350-like
d2ikbd_	d2ikba1	162	1.64	86	d.2.1.9	NMB1012-like
d2jdqe_	d2jdqd_	63	1.28	86	d.361.1.1	PB2 C-terminal domain-like
d4j2jc_	d4j2jb_	113	1.44	87	b.145.1.1	AXH domain
d1zvpc1	d1zvpb1	66	1.26	88	d.58.18.9	VC0802-like
d2q4fa_	d1wn0c_	135	1.10	88	a.24.10.2	Phosphorelay protein-like
d3t30h_	d3t30e_	90	1.40	89	b.121.3.0	automated matches
d1zrsb2	d1zrsa2	152	1.70	90	c.23.16.7	LD-carboxypeptidase A N-terminal domain-like
d2auab1	d2auaa1	188	1.36	93	d.166.1.6	BC2332-like
d2geca1	d2c86b_	131	1.00	94	b.148.1.1	Coronavirus RNA-binding domain
d2z16a_	d1ea3b_	149	1.06	95	a.95.1.1	Influenza virus matrix protein M1
d3f08b_	d3cnwa1	138	1.17	96	d.129.3.8	Atu1531-like
d1qqrd_	d1c4pa_	135	1.59	97	d.15.5.1	Staphylokinase/streptokinase
d2j70a_	d2j6yb_	79	0.46	97	a.186.1.2	Phosphoserine phosphatase RsbU, N-terminal domain
d2aaja1	d2aaga_	129	0.26	98	d.80.1.6	MSAD-like
d2c1ya2	d2c0fa2	121	0.69	98	c.47.1.7	ERP29 N domain-like
d3gjoc_	d3gjob_	63	1.08	98	a.245.1.1	EB1 dimerisation domain-like
d1fc9a3	d1fc6a3	92	0.66	99	b.36.1.3	Tail specific protease PDZ domain
d1l6pa_	d1jzdc_	117	1.17	99	b.1.17.1	Thiol:disulfide interchange N-terminal domain (DsbD-alpha)
d2ao9c_	d2ao9a1	116	0.48	99	a.4.1.17	Nanomeric phage protein-like
d2aunb1	d2auma1	156	0.58	99	c.8.10.1	LD-carboxypeptidase A C-terminal domain-like
d3nj5a_	d2wrya_	150	0.70	99	b.42.1.0	automated matches
d3qvga_	d3pc7a_	81	1.52	99	c.15.1.2	DNA ligase
d1dj8e_	d1bg8b_	76	0.71	100	a.57.1.1	Protein HNS-dependent expression A; HdeA
d1mg7b1	d1mg7a1	164	0.18	100	d.14.1.6	Early switch protein XOL-1, N-terminal domain

Table S3 (continue): Representative SCOP families.

ID1	ID2	Length*	E_r**	Seq Id (%)	Family id	Description
d1r0vd3	d1r0va3	75	0.24	100	d.75.1.1	tRNA-intron endonuclease N-terminal domain-like
d1t1jb_	d1t1ja_	119	0.64	100	c.23.14.2	Hypothetical protein PA1492
d1ufbd_	d1ufbb_	127	0.19	100	a.24.16.3	HEPN domain
d1v8hb_	d1v8ha1	106	0.54	100	b.1.18.19	SoxZ-like
d1w94b1	d1w94a1	149	0.44	100	c.51.1.2	Brix domain
d1ytlc_	d1ytla1	157	0.39	100	c.31.1.6	ACDE2-like
d2cxdb_	d2cwya1	94	0.56	100	a.246.2.1	TTHA0068-like
d2dlbb_	d2dlba1	70	0.51	100	b.174.1.1	YopT-like
d2dstb_	d2dsta1	122	0.76	100	c.69.1.39	TTHA1544-like
d2f9hb_	d2f9ha1	119	0.53	100	b.161.1.1	PTSIIA/GutA-like
d2fhza1	d2dfxi1	83	0.20	100	d.311.1.1	ImmE5-like
d3gefc_	d3gefb_	117	0.38	100	b.1.16.1	Lamin A/C globular tail domain

* Alignment length.

** Residual root-mean square error.

References

Bloom, Jesse D. 2015. "Software for the analysis and visualization of deep mutational scanning data." *BMC bioinformatics* 16:168.

Drummond, D Allan and Claus O Wilke. 2008. "Mistranslation-induced protein misfolding as a dominant constraint on coding-sequence evolution." *Cell* 134:341–352.

Fersht, Alan et al. 1999. *Structure and mechanism in protein science: a guide to enzyme catalysis and protein folding*. Macmillan.

Gronenborn, Angela M, David R Filpula, Nina Z Essig, Aniruddha Achari, Marc Whitlow, Paul T Wingfield, and G Marius Clore. 1991. "A novel, highly stable fold of the immunoglobulin binding domain of streptococcal protein G." *Science(Washington)* 253:657–661.

Malakauskas, Sandra M and Stephen L Mayo. 1998. "Design, structure and stability of a hyperthermophilic protein variant." *Nature structural biology* 5:470–475.

McCallister, Erika L, Eric Alm, and David Baker. 2000. "Critical role of [beta]-hairpin formation in protein G folding." *Nature Structural & Molecular Biology* 7:669.

Soskine, Misha and Dan S Tawfik. 2010. "Mutational effects and the evolution of new protein functions." *Nature Reviews Genetics* 11:572.
